# Targeting Copper in Cancer: Chelators to Counteract Cuproplasia and Ionophores to Promote Cuproptosis

**DOI:** 10.1021/acs.jmedchem.6c00884

**Published:** 2026-07-11

**Authors:** Sabrina Castellano, Alessandra Tosco, Ciro Milite, Monica Viviano, Tiziano Marzo, Sabrina Taliani, Gianluca Sbardella

**Affiliations:** † Department of Pharmacy, 19028 University of Salerno, via Giovanni Paolo II 132, I-84084 Fisciano, Salerno, Italy; ‡ Department of Pharmacy, 9310 University of Pisa, via Bonanno Pisano 6, I-56126 Pisa, Italy

## Abstract

Precise copper homeostasis is vital for cellular physiology, and its dysregulation is closely linked to several pathological conditions. Within oncology, copper exerts a dual role. On the one hand, cellular copper is essential for promoting tumor growth and progression (cuproplasia). On the other hand, the accumulation of intracellular copper can induce cytotoxicity and regulated cell death (cuproptosis). Consequently, therapeutic strategies that either utilize chelators to inhibit cuproplasia or employ ionophores to induce cuproptosis are emerging as innovative anticancer approaches. Despite several compounds entering clinical trials, copper-targeted drug discovery remains underexploited due to incompletely elucidated molecular pathways and a lack of ligands with highly specific mechanisms of action. This Perspective provides a summary of the role of copper in cancer and furnishes an updated overview of ligands used to modulate cellular copper levels, detailing their structural, chemical, and biological properties, while outlining future directions for drug discovery in this field.

## Graphical abstract

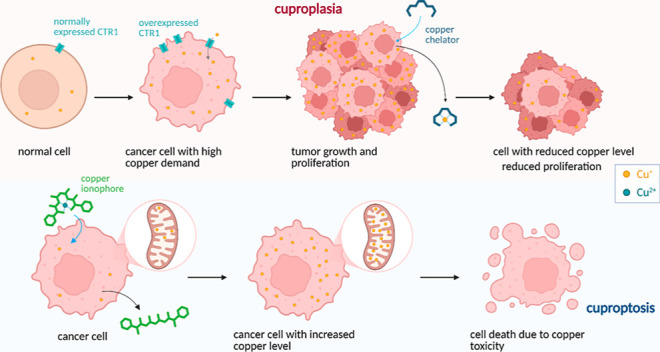

## 1 Significance

- This Perspective summarizes the dual role of copper in cancer, giving an overview on the mechanisms of tumor growth (cuproplasia) and copper-induced cell death (cuproptosis).
- It provides an updated overview of copper-modulating ligands, detailing the structural, chemical, and biological properties of chelators and ionophores.
- Finally, it outlines current challenges and future directions in the design of next-generation anticancer therapies targeting copper.

## 2 Introduction

Metal complexes have played a pivotal role in cancer chemotherapy since the landmark discovery of cisplatin. Platinum-based compounds are highly effective due to their ability to form covalent bonds with DNA, leading to DNA adducts and cross-links. However, despite their widespread use in the treatment of various cancers, clinically approved platinum-based drugs have several drawbacks, including high systemic and acute toxicity. Additionally, they display limited or no activity in relapsed disease due to the frequent development of resistance.^1^ These limitations prompted active research into exploring other metal-based complexes with greater anticancer potential and fewer adverse effects. Compounds featuring non-endogenous metals like platinum, palladium, gold or ruthenium exert their anticancer properties through various mechanisms. These include, for example, DNA binding and protein metalation, this latter resulting in the modulation or inhibition of a wide range of protein targets, including enzymes, ultimately leading to cancer cell death.^2,3^ While these metals show promising potential to overcome drug resistance, they still face challenges related to stability and targeted delivery.

Recently, the field of anticancer metal complexes has been expanded to explore the potential of complexes based on endogenous metals like zinc, iron and copper. These metals are essential for life as they play crucial roles in biological processes, but they can be harmful if present in excess. Cancer cells often exhibit dysregulated essential metal homeostasis, making them particularly susceptible to metal-based intervention. Several endogenous-metal complexes have been developed as putative potent antineoplastic agents by exploiting the endogenous regulatory pathways of essential trace elements to achieve high selectivity and reduced systemic toxicity compared to xenobiotic metallodrugs. The therapeutic efficacy of zinc complexes is primarily driven by the disruption of cellular homeostasis, involving the inhibition of zinc-dependent enzymes, the displacement of native ions in DNA-binding proteins, and the induction of mitochondrial-mediated apoptosis via oxidative stress.^4^ Iron, an essential micronutrient for numerous physiological processes, can lead to iron-mediated oxidative stress and ferroptosis, an iron-dependent form of regulated cell death. Although earlier observations of iron-dependent, nonapoptotic cell death date back several decades, the term ″ferroptosis″ was introduced only in 2012 to distinguish this specific iron-dependent process from other cell death pathways, such as apoptosis, unregulated necrosis, and necroptosis.^5^ Mechanistically, ferroptosis is driven by redox imbalance that triggers an excessive buildup of reactive oxygen species (ROS) mediated by iron-dependent Fenton reaction. This process promotes lipid peroxidation and results in oxidative damage to cellular membranes. When cellular antioxidant defenses are overwhelmed, ferroptotic cell death occurs. Therefore, disrupting redox homeostasis by high intracellular levels of iron provides new insights for cancer therapy.^6^

In this context, modulating cellular copper levels with copper-binding compounds has also emerged as a promising strategy for the development of new anticancer drugs. From the pharmacological point of view, copper functions as a double-edged sword within cells: it can drive tumor development and progression through several molecular mechanisms or, conversely, inhibit tumor growth and promote cell death.^7^ However, the precise molecular mechanisms underlying the role of copper in cancer cells are not completely understood and further studies are needed to delineate the correlation between copper imbalances and specific cancer phenotypes. In recent years, fundamental progress has been made and the introduction of the terms “cuproplasia”^8^ and “cuproptosis”^9^ witnesses these advances. In parallel, the development of copper ligands for targeting cuproplasia and cuproptosis has emerged as a promising and innovative strategy for anticancer therapies. Accordingly, copper-binding compounds are developed as either copper chelators or ionophores to exploit these opposing mechanisms. While some copper ligands are under evaluation in clinical trials, either alone or as adjuvants in chemotherapy or radiotherapy, the field faces several challenges. Most compounds with putative anticancer activity were identified by serendipity or repositioning approaches rather than rational design, meaning that the structure–activity relationships (SARs) required for optimization are missing. Consequently, the exploration into the anticancer efficacy of copper-based compounds has lagged behind.

The rapid expansion of research into copper-regulated cell death has necessitated a re-evaluation of the chemical and biological profiles of copper-binding small molecules. While the general roles of cuproptosis and cuproplasia have been widely discussed in recent literature, this Perspective summarizes the role of copper in cancer cells and focuses specifically on the chemical and physical properties that dictate the efficacy of these agents. Herein, we present a curated analysis of the most promising copper chelators and ionophores, emphasizing their SARs and *in vitro* characterization. By providing this detailed chemical-structural viewpoint, we aim to offer a specialized framework to assist medicinal chemists in the rational design and chemical and biological evaluation of new molecules targeting copper pathways in cancer.

## 3 Systemic and Intracellular Copper Homeostasis

Copper is an essential metal that plays a crucial role in biological processes; therefore, its concentration must be tightly regulated to maintain cellular homeostasis. In biological systems, copper exist in two oxidation states, Cu­(I) and Cu­(II) and most intracellular copper is bound to proteins. Indeed, free cytosolic copper is present only in traces underlining the pivotal role of trafficking systems in systemic and intracellular copper distribution (Figure 1).^10–13^

**Figure 1:**
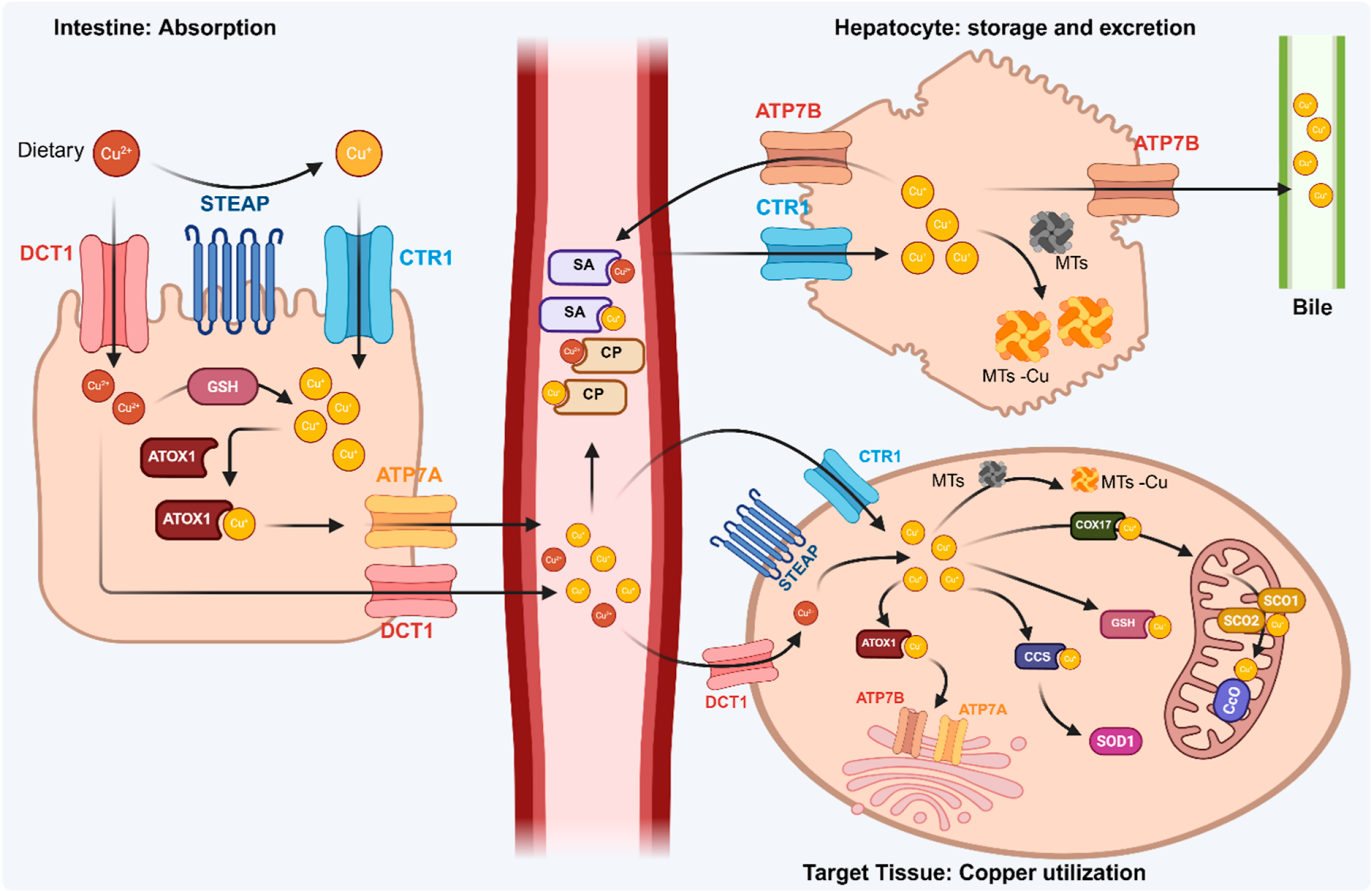
Systemic and intracellular copper homeostasis. (Created in BioRender (2026) https://BioRender.com/htvryex). Dietary copper is acquired as Cu­(II) which can be directly absorbed by enterocytes via the nonspecific divalent metal transporter DCT1 or reduced to Cu­(I) by the metalloreductase STEAP for uptake via the high-affinity transporter CTR1. Within the intestinal cytosol, Cu­(II) is partially reduced to Cu­(I) by GSH and subsequently bound by the chaperone ATOX1. Copper is then transported into the bloodstream through ATP7A or as Cu­(II) via basolateral DCT1. In systemic circulation, copper binds to carrier proteins such as SA and CP to reach target organs. In the liver, hepatocytes uptake copper primarily via CTR1; once internalized, copper is either stored in MTs or trafficked to ATP7B for biliary excretion or redistribution. In other tissues, copper enters the cell through CTR1, STEAP, and DCT1. Intracellular copper is distributed to various functional sites by specific chaperones. ATOX1 delivers copper to ATP7A/ATP7B in the TGN for enzyme synthesis; CCS delivers it to SOD1 to manage oxidative stress; COX17, in coordination with SCO1 and SCO2, facilitates copper delivery to CcO within the mitochondria to support the electron transport chain. Abbreviations: *DCT1*: Divalent-cation transporter 1, *STEAP*: Six-transmembrane epithelial antigen of the prostate, *CTR1*: Copper transport protein 1, *GSH*: Glutathione, *ATOX1*: Antioxidant 1 copper chaperone, *ATP7A/B*: Copper-transporting P-type ATPase α/β, *CP*: Ceruloplasmin, *SA*: Serum albumin, *MTs:* Metallothioneins, *TGN*: Trans-Golgi network, *CCS*: Copper chaperone for superoxide dismutase 1, *SOD1*: Superoxide dismutase 1, *CcO*: Cytochrome *c* oxidase, *COX17*: Cytochrome *c* oxidase copper chaperone, *SCO1/2*: Synthesis of cytochrome *c* oxidase 1 and 2.

Dietary copper is absorbed in the small intestine as Cu­(II) via Divalent-Cation Transporter 1, (DCT1, also called Divalent Metal Transporter 1, DMT1),^14^ but mainly after the reduction of Cu­(II) to Cu­(I) by the Six-Transmembrane Epithelial Antigen of the Prostate (STEAP) metalloreductases,^15^ via Copper Transporter 1 (CTR1), which is highly selective for Cu­(I).^16^ Once inside intestinal cells, Cu­(I) is transferred by the copper chaperone ATOX1 (Antioxidant Protein 1) to the copper-transporting P-type ATPase (ATP7A) for export into the bloodstream.^17^ Glutathione (GSH) also contributes to copper homeostasis facilitating its transfer to chaperones.^18^ Upon entering systemic circulation, copper binds to carrier proteins, mainly ceruloplasmin (CP) and serum albumin (SA) to reach target tissues. The liver acts as the central hub for copper distribution, storage and excretion.^19^ Hepatocytes acquire copper via CTR1 and direct it toward storage by metallothioneins (MTs),^20^ systemic distribution through the copper-transporting P-type ATPases β (ATP7B),^17^ or biliary excretion, the primary route for copper elimination.

Within target cells, copper is handled by a fine-tuned trafficking machinery that includes transporters, chaperones, storage proteins and metalloenzymes.^11,12^ It can be stored by MTs, bound to GSH or delivered by specific copper chaperones such as ATOX1, Copper Chaperone for Superoxide dismutase 1 (CCS) and Cytochrome *c* Oxidase copper chaperone (COX17) to distinct intracellular destinations. ATP7A and ATP7B mediate copper translocation within the Trans-Golgi Network (TGN) to support the biosynthesis of copper-dependent enzymes.^21^ COX17 and other specialized mitochondrial metallochaperones, such as Synthesis of Cytochrome *c* Oxidase 1 and 2 (SCO1 and SCO2), facilitate copper transport to mitochondria and its incorporation into Cytochrome *c* Oxidase (CcO, also known as complex IV), a key component of mitochondrial respiration.^22^ Furthermore, CCS delivers copper to Superoxide Dismutase 1 (SOD1), an antioxidant enzyme that protects cells from oxidative stress by scavenging superoxide radicals.^23^ Notably, SOD1 and ATOX1 ^24^ are overexpressed in cisplatin-resistant cancer cells, owing to their pro-survival functions.^25^

Because of its central metabolic role, copper dyshomeostasis is associated with several pathological conditions. Menkes disease (MD) is a rare and fatal X-linked genetic disorder caused by a systemic copper deficiency resulting from mutations in the ATP7A transporter (also known as Menkes’ protein).^26^ In contrast, Wilson disease (WD) is a rare genetic disorder caused by mutations in the ATP7B transporter, leading to toxic copper accumulation, particularly in liver and brain.^27^ Altered copper metabolism has also been implicated in Alzheimer’s disease (AD), although the underlying mechanisms remain controversial.^28,29^ Remarkably, recent years have witnessed a growing interest in the role of copper in the onset and progression of cancer.

## 4 Role of Copper in Cancer

Recent studies have identified imbalances in copper homeostasis across various cancers. It is now well established that copper plays a dual role in cancer biology: on the one hand, copper overload can promote tumor growth and proliferation (cuproplasia^8^), on the other hand, excessive intracellular copper accumulation triggers a specific form of regulated cell death (cuproptosis^9^). Therefore, modulation of copper levels has garnered increasing interest as an innovative approach to cancer therapy. Indeed, in the past few years, the number of studies aimed at elucidating the molecular mechanisms underlying copper-dependent tumor processes and at developing copper-targeted therapeutic approaches has increased dramatically (Figure 2). Although important advances have been made in understanding the roles of copper in cancer, several limitations and challenges persist in uncovering how copper dysregulation affects cancer cells. Subsections [Sec sec4.1] and 4.2 offer a description of the recently characterized processes of cuproplasia and cuproptosis, while the main signaling pathways and key regulators involved in each are summarized in Table 1.

**Figure 2:**
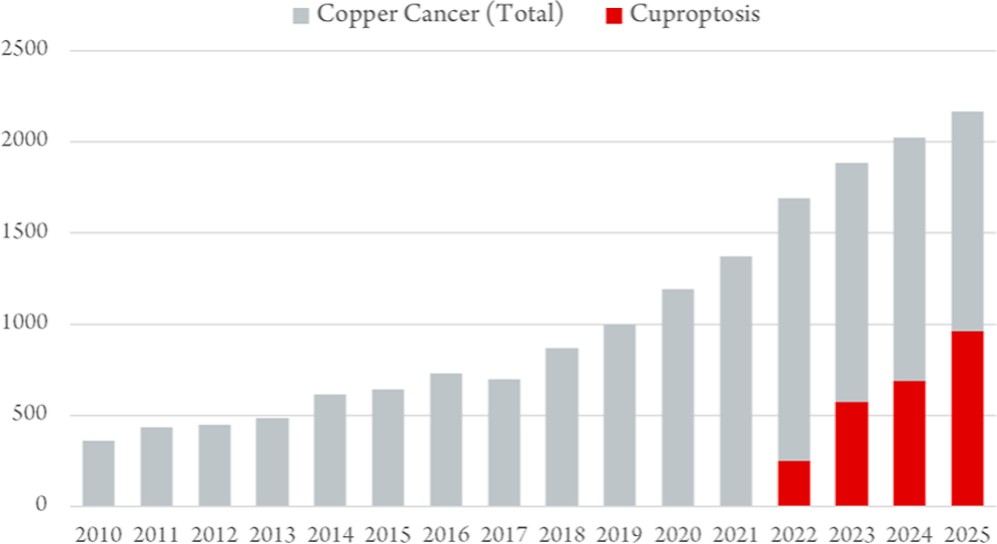
Evolution of copper-related cancer research (2010–2025). The graph shows the steady growth of general studies on copper in oncology (gray bars), followed by an exponential growth in publications specifically focused on cuproptosis (red bars) starting in 2022. Data source: Scopus.

**Table 1:** Comparative Features of Cuproplasia and Cuproptosis

feature	cuproplasia	cuproptosis
key triggers	increased copper uptake, metabolic rewiring, oncogenic signaling	acute copper overload, copper ionophores, impaired detoxification (e.g., ATP7A/B loss)
key copper-binding targets	Cu chaperones (ATOX1, CCS), MEK1/2, PI3K, transcription factors (SP1, HIF1α)	mitochondrial lipoylated proteins (DLAT, DLST), Fe–S cluster proteins, FDX1
signaling pathways	MAPK, PI3K/AKT, MEK/ERK, HIF1α, angiogenic pathways	proteotoxic stress, lipoylated protein aggregation, loss of Fe–S clusters
metabolic effect	aerobic glycolysis (Warburg effect), increased glutathione (GSH) to buffer copper	oxidative phosphorylation (OXPHOS) dependency, reduced GSH, elevated reactive oxygen species (ROS)
main cellular outcome	enhanced proliferation, survival, angiogenesis, and metastasis, therapy resistance	proteotoxic stress, mitochondrial dysfunction, and cell death

### 4.1 Copper Role in Tumor Development and Progression. Cuproplasia

For decades, it has been well established that cancer cells require higher copper levels than their normal counterparts.^30^ Multiple studies have reported elevated copper concentration in *ex vivo* cancerous tissues and in the serum of animal models and patients with different types of cancers such as breast,^31^ prostate,^32^ lung,^33^ and brain.^34^ Consistent with this increased demand, the expression of CTR1, the main system for copper uptake, is upregulated in several tumor samples and this overexpression has been associated with poor prognosis in patients with breast invasive carcinoma, lower grade glioma, mesothelioma, and skin cutaneous melanoma.^35^ In addition, copper and copper-containing molecules have been shown to exert pro-angiogenic effects in animal models while mild copper deficiency reduces angiogenesis, further supporting the role of copper in tumor growth and invasion.^36–38^ Although the detailed molecular mechanisms linking cellular copper accumulation to tumorigenesis have not been fully elucidated, recent research has significantly expanded the understanding of copper homeostasis in cancer cells. These studies have shed light on the effects of copper on a wide range of enzymatic activities and complex signaling pathways. Notably, a 2022 paper published in *Nature* introduced the term “cuproplasia” to define the copper dependent cell growth and proliferation.^8^ Here, we briefly summarize the primary molecular mechanisms proposed to underlie copper-driven tumor development (Figure 3). A detailed description of cuproplasia, which is beyond the scope of this perspective, could be found in excellent reviews recently published.^39,40^

**Figure 3:**
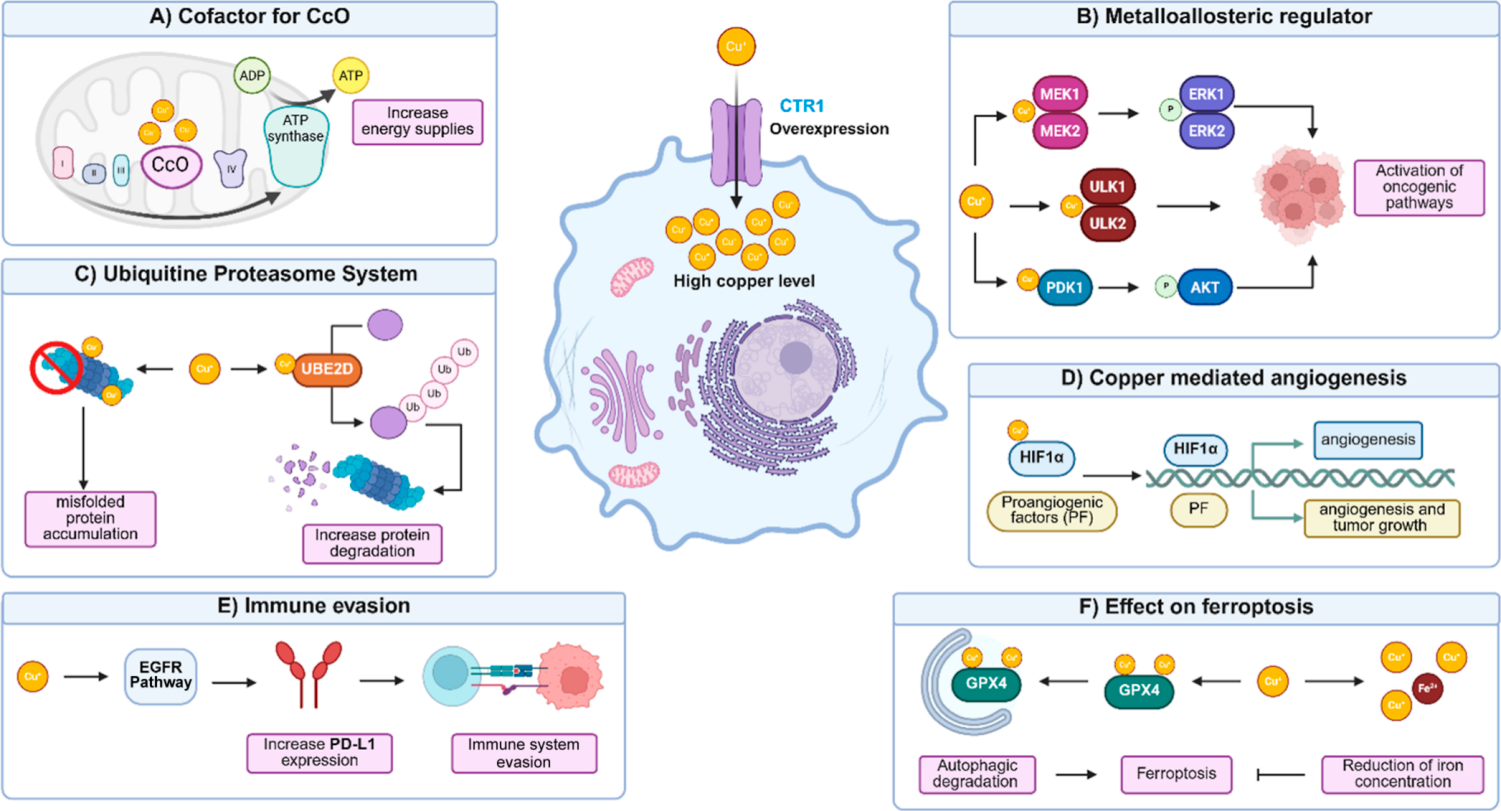
Main mechanisms in copper-dependent tumor development (cuproplasia) (Created in BioRender. (2026) https://BioRender.com/sdqo9d4). (A) Cofactor for CcO: copper serves as an essential cofactor for mitochondrial CcO, enhancing oxidative phosphorylation and ATP production to meet the high energy demands of cancer cells. (B) Metalloallosteric regulator: copper acts as a signal transducer by binding and modulating the activity of key kinases. It activates the MEK1/MEK2 – ERK1/ERK2 pathway, the PDK1 – AKT signaling axis, and the ULK1/ULK2 complex driving oncogenic growth and autophagy. (C) Ubiquitin Proteasome System: copper exerts a dual role by either promoting the degradation of tumor suppressors through the allosteric activation of the E2 ubiquitin-conjugating enzyme UBE2D, or by inhibiting the proteasome, leading to the accumulation of misfolded proteins. (D) Copper-mediated angiogenesis: copper promotes the stabilization of HIF1α and its binding to target genes, together with other PF, to stimulate tumor vascularization. (E) Immune evasion: copper levels influence the EGFR signaling pathway to upregulate PD-L1 expression, allowing cancer cells to evade the immune system. (F) Effect on ferroptosis: copper exhibits a complex role in ferroptosis as it can promote it via the autophagic degradation of GPX4 or inhibit it by modulating intracellular iron concentrations and oxidative stress responses. Abbreviations: *CTR1*: copper transporter 1, *CcO*: cytochrome *c* oxidase, *ATP*: adenosine triphosphate, *ADP*: adenosine diphosphate, *MEK1/2*: mitogen-activated protein kinase kinases 1 and 2, *ERK1/2*: extracellular signal-regulated kinases 1 and 2, *ULK1/2*: Unc-51 like autophagy activating kinase 1 and 2, *PDK1*: 3-phosphoinositide-dependent protein kinase 1, *AKT*: protein kinase B, *UBE2D*: ubiquitin-conjugating enzyme E2 D, *Ub:* ubiquitin, *HIF1*α: hypoxia-inducible factor 1-alpha, *PF:* proangiogenic factors, *EGFR:* epidermal growth factor receptor, *PD-L1:* programmed death-ligand 1, *GPX4:* glutathione peroxidase 4.

#### 4.1.1 Copper as a Cofactor of Cytochrome *c* Oxidase

As cancer cells have greater energetic demands compared with nonproliferating cells, they exhibit a higher requirement for copper as an essential cofactor of mitochondrial CcO. Copper availability is therefore critical for efficient mitochondrial respiration and ATP production, processes that sustain the high metabolic activity and rapid proliferation of tumor cells.^41^

#### 4.1.2 Copper as a Metalloallosteric Regulator

For decades, metals have been considered merely cofactors required for protein function. However, the recent expansion of traditional metallobiochemistry has introduced the concept of metalloallosterism, defined as the interaction of metal ions with specific protein binding sites to modulate their activity. In this context, copper has shown the ability to act as both a negative and positive allosteric regulator of protein activity, thereby influencing fundamental cellular pathways.^42^ Several studies have highlighted the role of copper as a modulator of kinase signaling. When the cytoplasmic concentration rises, copper can directly bind and activate Mitogen-activated protein kinase kinases 1 and 2 (MEK1/2), which, in turn, enhances Extracellular signal-Regulated Kinases 1 and 2 (ERK1/2) phosphorylation and triggers the MAPK/ERK signaling cascade that drives malignant transformation.^43^ Additionally, copper can bind to 3-Phosphoinositide Dependent protein Kinase 1 (PDK1) promoting its interaction with and consequent activation of AKT, a key kinase involved in cell proliferation and survival.^44^ Finally, through direct interaction, copper activate Unc-51-Like autophagy-activating Kinase 1 and 2 (ULK1 and ULK2). These key kinases trigger autophagy in mammals, thereby supporting tumor survival and proliferation in established cancers.^45^

#### 4.1.3 Copper and Ubiquitin-Proteasome System

Copper plays an intricate, dual role in the UPS pathway, acting as both an activator and an inhibitor.^46^ On the one hand, through allosteric activation of E2 Ubiquitin-conjugating Enzyme D (UBE2D), copper can increase polyubiquitination and accelerate the degradation of key regulatory proteins, including the tumor suppressor p53. On the other hand, elevated copper levels can impair proteasome function, resulting in the accumulation of misfolded proteins.^47^

#### 4.1.4 Copper Mediated Angiogenesis

As previously mentioned, the role of copper in promoting angiogenesis is well established, though the precise molecular mechanisms remain under investigation. Elevated copper levels have been correlated with increased expression and transcriptional activity of proangiogenic factors, thereby promoting angiogenesis and tumor growth.^38^ In addition, copper enhances the binding of Hypoxia-Inducible Factor 1α (HIF1α) to hypoxia-responsive elements in its target genes, further amplifying the angiogenic process.^48^

#### 4.1.5 Copper-Driven Cancer Immune Evasion

Recent evidence indicates that increased copper levels in certain cancer cells enhance the expression of Programmed Death Ligand 1 (PD-L1), a key immune-checkpoint protein exploited by cancer cells to elude the immune response. PD-L1 upregulation is often induced by the activation of oncogenic pathways including the Epidermal Growth Factor Receptor (EGFR) signaling pathway. Moreover, in tumors such as neuroblastoma, a statistically significant positive correlation has been observed between the expression of the copper transporter CTR1 and PD-L1, further supporting the link between copper homeostasis and immune escape mechanisms.^49^

#### 4.1.6 Inhibition of Ferroptosis

The enzyme Glutathione Peroxidase IV (GPX4) serves as a key inhibitor of ferroptosis by reducing lipid peroxides.^50^ Copper can exert a dual role in ferroptosis, as it can both promote and inhibit the process depending on the cellular context. Several studies indicate that copper overload promotes ferroptosis through several mechanisms including the autophagic degradation of GPX4.^51^ Conversely, in other contexts, copper can inhibit lipid peroxidation and ferroptosis by reducing intracellular iron concentration and promoting HIF1α stabilization.^52^

Taken together, these findings indicate that many cancer cells increase copper uptake by upregulating the expression of CTR1, and the concurrent enhancement of intracellular copper levels promotes cell growth and differentiation. Cuproplasia arises from the multifaceted impact of copper on several cellular processes. These include increased energy production, stimulation of angiogenesis, autophagy and immune evasion, as well as modulation of proteasome activity and impairment of tumor-suppressor protein function or accumulation of misfolded proteins, and activation of oncogenic signaling pathways. Accordingly, cuproplasia could be suppressed by reducing cellular copper levels; indeed, copper chelators have been proposed as potential anticancer agents, either alone or in combination with chemotherapy, immunotherapy or radiation (see below).

### 4.2 Copper Role in Inhibiting Tumor Growth and Promoting Cell Death. Cuproptosis

The cytotoxic activity of metal-based agents has been exploited for disease treatment since ancient times. The discovery of the anticancer properties of cisplatin in the 1960s marked the beginning of modern metal-based cancer therapy. Following the FDA approval of cisplatin for the treatment of specific malignancies in 1978, research efforts expanded to investigate other metals, such as ruthenium, gold, palladium, iridium, copper, iron, and zinc, aiming to exploit their cytotoxic potential and eventually overcome resistance and toxicity issues associated with platinum-based drugs. In this context, the relationship between copper overload and cell death has been a focus of investigation for several decades.^53^ Based on these considerations, numerous copper complexes have been synthesized and evaluated as putative anticancer agents.^54^ However, the detailed molecular pathways driving their antitumor activity have not been fully elucidated leading over the years to the proposal of several mechanisms of action (Figure 4).

**Figure 4:**
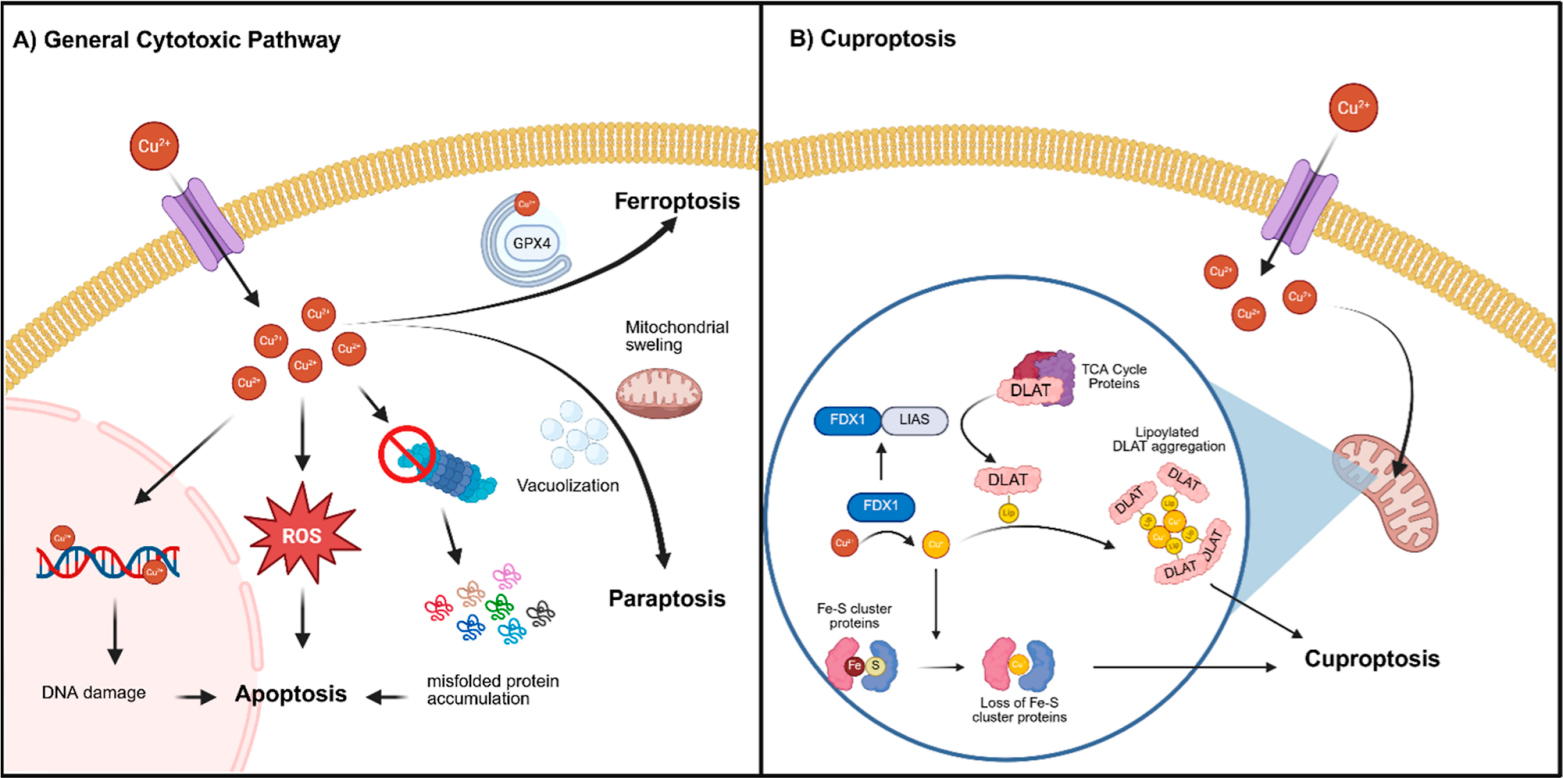
Main mechanisms of copper-induced cell death. (Created in BioRender (2026) https://BioRender.com/blnbqap). (A) General Cytotoxic Pathways. Intracellular copper overload causes cell death through several pathways. Apoptosis is induced by direct DNA damage, oxidative stress or inhibition of the UPS. Additionally, copper induces ferroptosis, by promoting the autophagy of GPX4, and paraptosis. (B) Cuproptosis. In the mitochondria, FDX1 generates Cu­(I) from Cu­(II). Cu­(I) binds to the lipoyl moiety of TCA cycle proteins, such as DLAT, causing toxic protein aggregation. This aggregation, combined with the loss of essential Fe–S cluster proteins, results in proteotoxic stress and subsequent cell death. Abbreviations: *ROS:* reactive oxygen species, *UPS:* ubiquitin-proteasome system, *GPX4:* glutathione peroxidase 4, *FDX1:* ferredoxin 1, *TCA:* tricarboxylic acid (cycle), *DLAT:* dihydrolipoamide S-acetyltransferase, *LIAS:* lipoic acid synthase, *Lip:* lipoyl moiety.

#### 4.2.1 Apoptotic Mechanisms

Early studies related the anticancer activity of copper complexes to the induction of apoptosis, triggered by several pathways.

##### 4.2.1.1 DNA Damage

Similar to the mechanism widely illustrated for cisplatin, copper, especially in its oxidized Cu­(II) form, exhibits high DNA binding affinity leading to DNA damage and subsequent activation of apoptotic pathways.^55^ Accordingly, a high number of copper complexes featuring different size, affinity, and geometry have been investigated as potential DNA-targeting metallodrugs.

##### 4.2.1.2 Oxidative Stress

Conventional interpretation has often attributed copper-induced cell death to oxidative stress. In analogy to the mechanism proposed for ferroptosis, copper can generate highly reactive hydroxyl radicals through the Fenton reaction. Therefore, several authors proposed the resulting accumulation of ROS as a trigger for the apoptotic process.^56^

##### 4.2.1.3 Inhibition of the Ubiquitin-Proteasome System

As previously mentioned, copper can both activate and inhibit proteasome function. Copper-mediated impairment of proteasome function, not related to ROS generation, was first described two decades ago.^57^ Cellular copper overload was shown to induce toxic accumulation of misfolded proteins triggering the apoptotic process. In addition, it was highlighted that copper complexes able to inhibit proteasome activity possess specific metal-binding properties that facilitate cellular uptake and subsequent intracellular release, enabling direct interaction with cellular targets like the proteasome. Indeed, this proteasome inhibitory activity can be prevented by stronger chelators in which copper is fully sequestered within the ligand framework.^46,57,58^

##### 4.2.1.4 Protein Regulation

Other studies have highlighted the ability of a group of copper complexes to inhibit topoisomerases^59^ and to amplify the activity of various proteins involved in apoptotic pathways, such as p53.^60^ These findings have expanded the array of potential biochemical targets underlying the proapoptotic properties of these compounds.

#### 4.2.2 Non-apoptotic Mechanisms

While early evidence suggested that copper-induced cytotoxicity was mainly mediated by apoptosis, in recent years, alternative molecular mechanisms have been disclosed.

##### 4.2.2.1 Induction of Ferroptosis

As discussed above, copper can promote cell death by inducing the autophagic degradation of GPX4 thereby promoting ferroptosis.^51,61^

##### 4.2.2.2 Induction of Paraptosis

Since 2006, several studies have pointed out that the cytotoxic potential of some copper complexes is not associated with apoptosis but rather to a paraptotic cell death mechanism.^62,63^ Paraptosis is a form of programmed cell death hallmarked by the swelling of mitochondria and endoplasmic reticulum, as well as massive vacuolization.^64^ In 2011, further evidence demonstrated that copper itself is responsible for the paraptotic cell death in susceptible cancer cells. Crucially, the authors proposed that copper complexes able to induce caspase-independent death behave as copper ionophores, molecules that reversibly bind copper and facilitate its transport across cellular membranes. These compounds favor intracellular copper accumulation thereby exacerbating its intrinsic toxicity.^65^

#### 4.2.3 Cuproptosis

Very recently, a distinct form of regulated cell death triggered by intracellular copper overload has been identified. In 2019, the pioneering work of the Tsvetkov and Golub group introduced a pivotal player in copper dependent cell death, the ferredoxin1 (FDX1).^66^ FDX1 is a mitochondrial iron–sulfur containing protein that mediates electron shuttling among multiple metabolic pathways. It plays essential roles in physiological processes such as Fe–S cluster biogenesis, steroid hormone biosynthesis and other cytochrome-P450-associated reactions, and overall mitochondrial function.^67^ Remarkably, FDX1 also acts as an upstream regulator of protein lipoylation,^68^ a post-translational modification involving the covalent attachment of lipoic acid to lysine residues of specific proteins.

In mammals, four key mitochondrial enzymes, primarily associated with the tricarboxylic acid cycle (TCA) are lipoylated.^69^ FDX1 directly binds to lipoic acid synthase (LIAS) providing the electrons necessary for LIAS-mediated lipoylation of intracellular proteins. A key target of this process is dihydrolipoamide S-acetyltransferase (DLAT), a component of the pyruvate dehydrogenase complex (PDC) that catalyzes the conversion of pyruvate to acetyl-CoA, a central step in cellular respiration. In their breakthrough 2019 study,^66^ the authors found that a metabolic shift toward mitochondrial oxidative phosphorylation, rather than glycolysis as an energy source, confers not only resistance to proteasome inhibitors but also sensitivity to the drug elesclomol (**1**, ES, Figure 5), a well-known Cu­(II) ionophore. Among approximately 4300 molecules tested, this property was shared only by disulfiram (**2**, DSF, Figure 5), another copper-binding compound.

**Figure 5:**
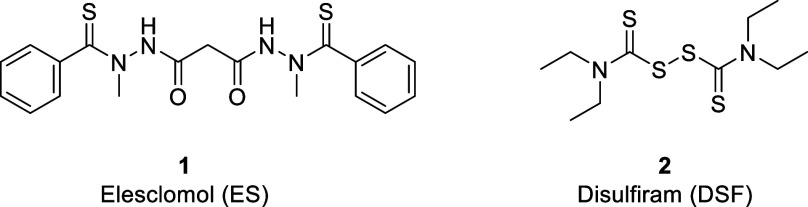
Structures of elesclomol (**1**) and disulfiram (**2**).

Notably, neither antioxidants nor inhibitors of apoptosis or ferroptosis were able to rescue **1**-induced cell death, whereas copper chelation did. Analysis of genomic features that correlate with **1** sensitivity identified FDX1 as the direct target of these copper ionophores. The study proposed that cell toxicity was driven by the interaction between the ionophore and FDX1 leading, therefore, to the inhibition of Fe–S cluster biosynthesis and mitochondrial function. In addition, they demonstrated that the Cu­(II) complex serves as a neo-substrate for reduced FDX1 leading to the generation of the more toxic Cu­(I) species. Since copper ionophores promote a distinctive form of cell death different than apoptosis or ferroptosis, the authors introduced the term “cuproptosis” to describe a copper-dependent mechanism of cell death. In 2022, the same group further elucidated the molecular mechanisms of copper-induced cell death and formally coined the term cuproptosis. The mechanism proposed proceeds as follows: copper ionophores bind Cu­(II) and facilitate its transport into mitochondria, where FDX1 binds Cu­(II) and reduces it to Cu­(I). The lipoyl moiety of TCA cycle proteins, particularly DLAT, serves as a direct Cu­(I) binder causing lipoylated protein aggregation and their toxic gain of function. The aggregation and subsequent loss of essential Fe–S cluster proteins contribute to impaired mitochondrial respiration and to proteotoxic stress, ultimately leading to cell death.^9^ Currently, DLAT aggregation in a copper-dependent manner is considered a hallmark of cuproptosis.^70,71^

Despite these significant advances, the complete identification of all cuproptosis effectors remains an active area of investigation. Recent studies have demonstrated that FDX1 is not strictly essential for the release of copper from other copper-ionophores; indeed, **1** itself can release Cu­(I) outside mitochondria in an FDX1-independent manner, making it available to nonmitochondrial cuproenzymes.^72^ Furthermore, FDX1-independent cell death induced by **1**-copper complex has been reported in astrocytes. Because astrocytes are not heavily reliant on mitochondrial respiration, the **1**-copper toxicity in these cells cannot be rescued by inhibitors of apoptosis or ferroptosis, whereas treatment with antioxidants is protective. Consequently, the author proposed that the mediator of **1**-copper effects in astrocytes is the oxidative stress via ROS production and lipid peroxidation, rather than the protein lipoylation pathway typical of classic cuproptosis.^73^

Emerging evidence also connects cuproptosis to epigenetic mechanisms. For example, in gastric cancer cells, where both FDX1 protein level and copper content are high, methyltransferase-like 16 (METTL16) plays an important role. METTL16 is an RNA methyltransferase responsible for *N* ^6^-methyladenosine deposition critical for tumorigenesis.^74^ It has been observed that this enzyme promotes FDX1 accumulation affecting its expression and consequently sustaining cellular susceptibility to cuproptosis. Consistently, METTL16 knockdown confers resistance to copper ionophores treatment.^75^

These findings have important implications for the development of copper complexes as anticancer agents. From a clinical perspective, tumor cells with high levels of FDX1 and lipoylated proteins are hypersensitive to cuproptosis. Therefore, the translation of promising preclinical results into clinical practice for copper ionophores should be accompanied by integrated multiomics analyses to identify predictive biomarkers for patient stratification.^70,76^ In parallel, the rational design and biological evaluation of copper complexes should take several aspects into account.

## 5 Copper Chelators as Cuproplasia Mitigators

Copper chelators are compounds capable of binding copper with high affinity and selectivity, therefore able to reduce its bioavailability and decrease its intracellular concentrations. Given the role of copper in tumorigenesis, copper chelators have been considered as putative anticancer drugs and have been exploited for advancing the understanding of copper’s role in promoting cell proliferation and growth. Mechanistically, copper chelators can inhibit several pathways involved in cuproplasia, including key angiogenic factors, the MAPK signaling pathway, and the immune checkpoint protein PD-L1 expression. Notably, their antiproliferative effect can be reversed by increasing the metal concentration. Penicillamine, polyamines and thiomolybdates (Figure 6), the copper chelators clinically used to lower elevated plasma copper levels in patients with WD, have been repositioned as anticancer agents. Currently, several Phase I/II clinical trials are ongoing to evaluate their safety and efficacy in combination with immunotherapy, chemotherapy, or radiotherapy for treating various malignancies. The preclinical and clinical status of these compounds is summarized in Table 2.

**Figure 6:**
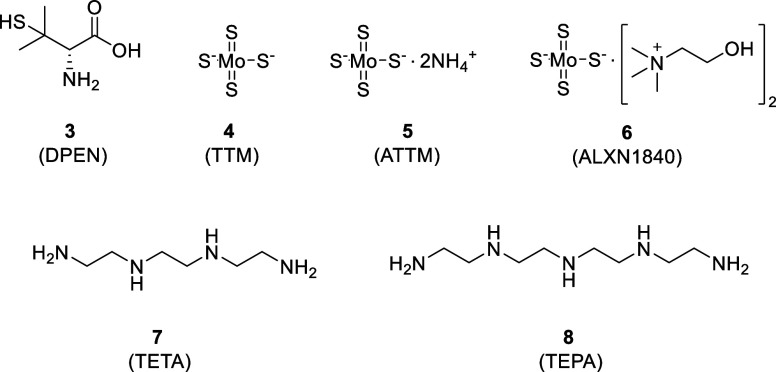
Structures of copper chelators **3–8**.

**Table 2:** Selected Preclinical and Clinical Studies on Copper Chelators Investigated as Cuproplasia-Mitigating Agents^a^

compound	selected preclinical studies (mouse)	selected clinical studies (phase, code)
**3** ^b^	oxaliplatin-resistant cervical cancer^78^	glioblastoma (phase II, NCT00003751)
**5** ^b^	breast cancer,^88^ thyroid cancer^90^	breast cancer (phase II, NCT00195091)
**7** ^b^	neuroblastoma^96^	epithelial ovarian cancer (phase I, NCT03480750)
**8**	neuroblastoma^49^	-
**9**	breast cancer^98^	-
**10**	triple-negative breast cancer^100^	-
**11**	triple-negative breast cancer^103^	-

a Selected preclinical studies are reported according to the disease context evaluated, whereas clinical studies are listed with trial phase and ClinicalTrials.gov identifier (NCT code).

b FDA-approved for Wilson’s disease (WD).

### 5.1 d-Penicillamine

d-Penicillamine (**3**, DPEN, Figure 6), an aminothiol metabolite of penicillin, chelates heavy metals, such as copper, lead, and mercury, forming soluble complexes that are excreted in the urine. Due to its systemic copper chelation properties, **3** has been used to treat WD since 1956.^77^ Notably, the l-enantiomer is associated with increased toxicity, and is not used clinically. Since copper depletion is related to the inhibition of tumor growth and angiogenesis, **3** has been repurposed in preclinical and clinical trials for cancer therapy, alone or in combination with other agents.^78,79^ Indeed, by binding to serum and tissue copper, **3** reduces the copper pool required by cancer cells for proliferation and migration.^80^ In addition, **3** exhibits pro-oxidant activity in certain cancer cells. As a matter of fact, thiol-containing compounds could protect cells against oxidative stress but can also be cytotoxic. In the presence of transition metals such as copper and iron, thiols oxidize to disulfides generating hydrogen peroxide (H_2_O_2_) and other ROS. The toxicity relies on several factors including metal presence, cell type, and the nature and concentration of the thiol. The selective cytotoxic effect of **3** against cancer cells, which can feature significantly higher levels of intracellular copper than normal cells, relies on its ability to bind Cu­(II) and reduce it to Cu­(I) generating ROS via one electron reduction of O_2_ to form O_2_^•‑^, which rapidly dismutates to form H_2_O_2_. The *in vitro* antiproliferative effect is completely inhibited by the presence of catalase and/or by Cu­(II) depletion, confirming that the cytotoxic effect of **3** results from copper mediated production of H_2_O_2_.^81,82^ However, further research is needed to effectively exploit these combinations in routine anticancer clinical practice. Furthermore, the clinical use of **3** is limited by its toxicity, which affects blood, kidneys, liver, and nervous system ranging from common side effects to severe, fatal issues.^83^

### 5.2 Tetrathiomolybdate Salts

Tetrathiomolybdate (**4**, TTM, Figure 6) is a tetrahedral molybdenum–sulfur anion with high copper chelating ability. The discovery of its anticopper effect dates back to the 1940s, when ruminants fed on forage with a high molybdenum concentration developed a disease later connected to copper deficiency. It was realized that, within the rumen, molybdenum was converted into **4**, which is a potent copper chelator. Due to its low toxicity and potential effectiveness in treating human copper overload disorders, the ammonium salt of **4** (**5**, ATTM, *Coprexa* ^,^ Figure 6) was proposed to treat WD.^84^ Preclinical and clinical studies indicate its efficacy for neurological and hepatic symptoms in WD, making it a promising alternative to approved chelation drugs.^85^ It has been proposed that **5** acts as both a chelator and a reductant, first reducing Cu­(II) to Cu­(I) and then binding the latter with higher binding affinity to form complexes. Because of a chemical reaction involved, the mechanism of **4** salts is distinct from that of other chelators such as **3**.^84^ As **5** yields stable tripartite complexes with copper and any proteins, it does not circulate in the free form in vivo. When administered orally, **5** binds immediately to dietary copper to produce a complex that is directly excreted into the bile without being absorbed in the gastrointestinal tract, therefore preventing dietary copper uptake. Alternatively, in the blood, **5** forms a stable tripartite complex with copper and albumin. In addition, **5** is able to remove intracellular metallothionein-bound copper from the liver.^86^ Despite the excellent copper-sequestering features of **5**, its therapeutic application for neurological symptoms in WD is limited by its negatively charged nature which prevents it from effectively crossing the blood–brain barrier. Moreover, the routine use of **5** was limited by its instability in aqueous solution. To address these issues, new formulations have been proposed. The choline salt of **4** was developed (**6**, bis-choline tetrathiomolybdate, ALXN1840 previously known as WTX-101 or ATN-224, Figure 6) as a more stable derivative with improved bioavailability^87^ and now in Phase III clinical trials for the treatment of WD. Due to the role of copper in tumors development, **4** salts were reproposed in preclinical and clinical studies as anticancer agents in patients for various malignancies, including kidney, breast, prostate, and hematological cancers.^88^ Mechanistically, **4** salts have the potential to counteract cuproplasia by inhibition of intracellular cuproenzymes, such as MEK1/2 kinase and SOD1, and the copper chaperone ATOX1.^86,89,90^

### 5.3 Polyamines

Because of the presence of properly linked multiple amine groups, polyamines act as multidentate ligands and are strong copper chelators.^91^ Triethylenetetramine (**7**, TETA, Figure 6), also known as trientine, is a potent and selective copper tetradentate chelator with a very high stability constant (log *K* = 20.4) for Cu­(II).^92^ Unlike **3**, which has only a modest effect on intestinal copper absorption, **7** functions as both systemic and dietary copper chelator. By forming a stable complex, **7** promotes the elimination of absorbed copper through urinary excretion while simultaneously inhibiting copper absorption within the intestinal tract. Indeed, because **7** has a low bioavailability (8–30%), it is poorly absorbed and remains available to bind dietary copper in the gut. In addition, **7** showed a superior safety profile compared to **3**. **7** dihydrochloride (trientine dihydrochloride, TETA-2HCl) has been approved for the treatment of WD since 1985. More recently, in 2022, the FDA approved the tetrahydrochloride salt of **7** (trientine tetrahydrochloride, TETA-4HCl, *Cuprior*) as new oral formulation.^93,94^

Similarly to other copper chelators, **7** has recently been proposed as a putative anticancer agent. Preliminary studies have shown encouraging results, both as monotherapy and in combination strategies to enhance the effectiveness of other cancer treatments. For example, **7** was investigated in ovarian cancer patients for enhancing the efficacy of carboplatin,^95^ and in preclinical neuroblastoma models as an adjuvant immunomodulatory agent to enhance the activity of anti-GD2 monoclonal antibodies.^96^

As an effective copper chelator, the molecular mechanism of **7**’s anticancer activity is primarily based on its ability to reduce cytoplasmatic copper levels in cancer cells, therefore counteracting cuproplasia. Specifically, **7** can inhibit several enzymes involved in carcinogenesis, such as SOD1, which is overexpressed in cisplatin-resistant cells.^25^ Additionally, it has been proven that **7** stabilize G-quadruplexes, which are specific structures found in the guanine-rich sequences of telomeres. By stabilizing these structures, **7** inhibits the activity of telomerase, a critical enzyme for maintaining telomere length in cancer cells.^97^

Tetraethylenepentamine (**8**, TEPA, Figure 6), a pentadentate Cu­(II) chelator and homologue of **7**, has been used in several preclinical models to evaluate the effect of copper chelation. **8** was successfully used to reveal the role of copper in modulating PD-L1 expressions in cancer cells and contributing to their immune evasion.^49^ These results have paved the way for repurposing **7**, already a clinically approved copper chelating agent, as an immunomodulatory agent to potentiate immunotherapy and improve responses in cancer patients.

### 5.4 New Strategies in the Development of Copper Chelators

The described copper chelators are FDA-approved medications for WD and, although evaluated in several clinical trials for cancer treatment, they have not yet achieved regulatory approval as anticancer agents. This could be ascribed to pharmacokinetic issues such as short half-life, water instability, and poor oral bioavailability as well as safety concerns. Therefore, current research is directed toward the development of new molecules and/or new strategies to harness the full potential of copper depletion in anticancer therapy.

One promising strategy is the use of polymeric copper chelators. It has been proposed that incorporating the small-molecule chelating group into water-soluble polymers can significantly improve their stability, plasma half-life, and safety profile. In a recent study, a hydroxyquinoline monomer, acting as a copper chelator, was copolymerized with a water-soluble poly­(ethylene glycol)­methyl ether methacrylate to generate high molecular weights (>22.000 Da) polymers of type **9** (Figure 7). These polymers proved to be highly effective and selective in chelating copper ions, and the high molecular weight confers them good metabolic stability. As far as cytotoxic activity is concerned, the polymers inhibited the proliferation of selected human tumor cell lines. Mechanistic investigations indicated that polymers of series **9** impair ATP production in cancer cells by depleting mitochondrial copper, thereby inhibiting the CcO activity. Furthermore, in *in vivo* experiments, they exhibited superior effectiveness in inhibiting lung metastasis compared with **4** salts.^98^

**Figure 7:**
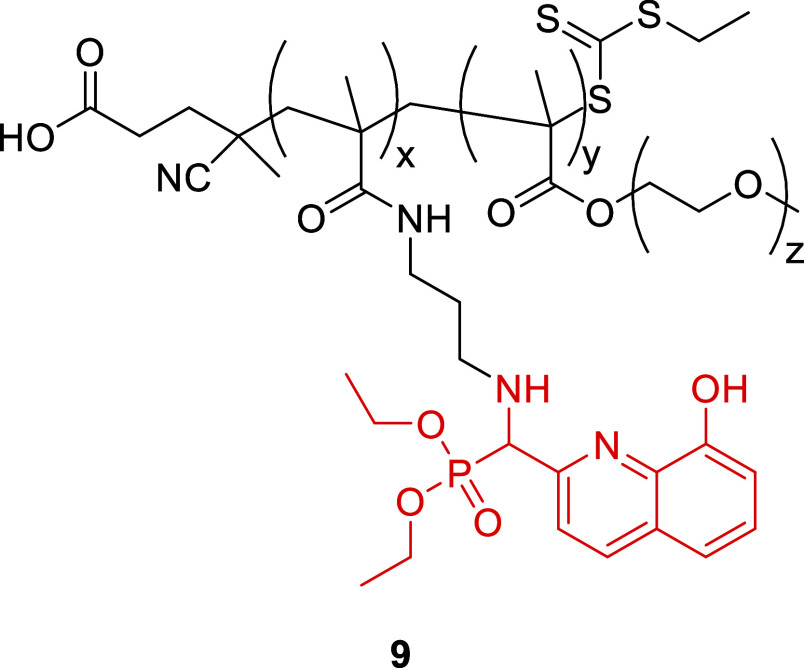
Structure of polymeric compounds of type **9**. Highlighted in red the copper chelating moiety.

With the aim of depleting copper levels selectively in the mitochondria, the bifunctional compound **10** was designed (Figure 8). It features a dipyridylamine, which acts as copper chelator moiety, tethered to a tricarbocyanine dye backbone. The dye, a hydrophobic highly delocalized cation that tends to localize to the mitochondria when incubated with cells, drives the compound’s specific accumulation within mitochondria. To improve plasma stability and enhance tumor delivery, **10** was incorporated within semiconducting polymer nanoparticles. In triple-negative breast cancer cells, nanoparticles of **10** exhibited cytotoxic effects related to irreversible structural change to mitochondrial morphology that causes reduced energy production and eventually cell death. This effect was confirmed to be copper-dependent, as the addition of exogenous copper prevented morphological damage. Moreover, in mouse models of triple-negative breast cancer, nanoparticles of **10** inhibited tumor growth and improved survival rates.^99,100^

**Figure 8:**
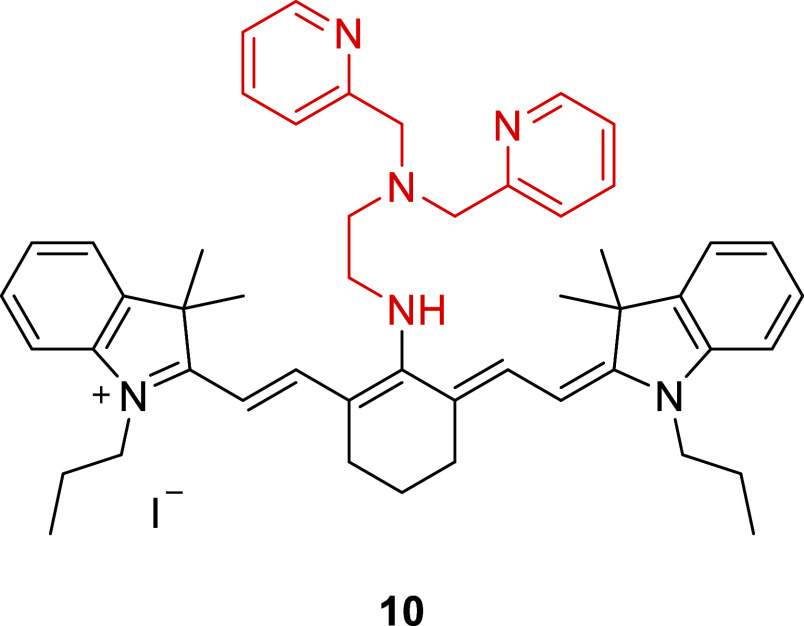
Structure of bifunctional compounds **10**. Highlighted in red the copper chelating moiety, in black the tricarbocyanine dye.

The relationship between resistance to radiation therapy and elevated intracellular copper in several cancers has paved the way for using copper chelators as adjuvants to improve the effectiveness of radiotherapy. Mechanistically, while radiotherapy exploits ferroptosis for its cytotoxic activity, it concurrently triggers adaptive responses by upregulating proteins that suppress ferroptosis, thereby contributing to radiotherapy resistance.^101^ Furthermore, as previously detailed, cuproplasia implies a role for copper in the inhibition of ferroptosis. This evidence stimulated the development of biomaterials designed to enhance the radiotherapy outcomes. Recently, a stimuli-responsive biomaterial based on oxime-urethane units was reported (Figure 9).

**Figure 9:**
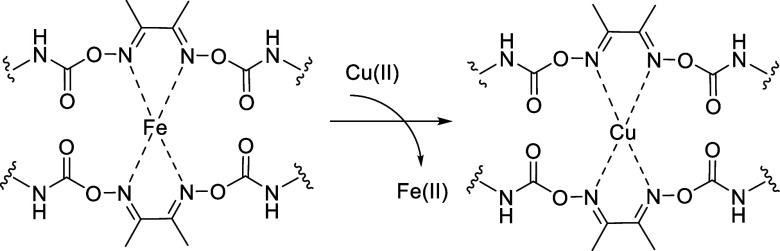
Structure of oxime–urethane ligands as iron/copper exchange system.

This platform, exploiting the differential coordination properties of oxime–urethane ligands for the two metal ions, coordinates iron, releasing it in the acidic microenvironment of cancers cells, and alongside chelating copper. By reversing tumor copper/iron homeostasis, the biomaterial promotes ferroptosis by releasing iron and prevents cuproplasia by chelating copper. Cellular assays confirmed that these nanodrugs sensitize tumor cells to radiotherapy through a ferroptosis-mediated mechanism.^102^

Another elegant strategy to exploit copper depletion for enhancing the effects of traditional therapeutic agents is the design of bifunctional molecules that incorporate both a copper chelator moiety and a well-validated anticancer agent. Researchers conjugated paclitaxel, a taxane compound that blocks the cell cycle by disrupting normal microtubule function, with 2,2-dipicolylamine, a copper chelator, through a disulfide bond. This yielded the redox-responsive prodrug **11** (Figure 10) which self-assembled with distearoyl phosphoethanolamine-PEG2000 to form stable nanoparticles, that showed efficient cellular uptake. In the specific intracellular redox environment, the disulfide bond can be either reduced to thiols or oxidized into hydrophilic sulfoxide/sulfone. These reactions promote the hydrolysis of adjacent ester bonds resulting in the concomitant release of free paclitaxel and the copper chelating moiety. In 4T1 cells and in a murine breast cancer model, **11** exhibited significantly enhanced efficacy compared with Taxol alone, confirming that copper depletion can induce cytotoxicity and potentiate chemotherapy.^103^

**Figure 10:**
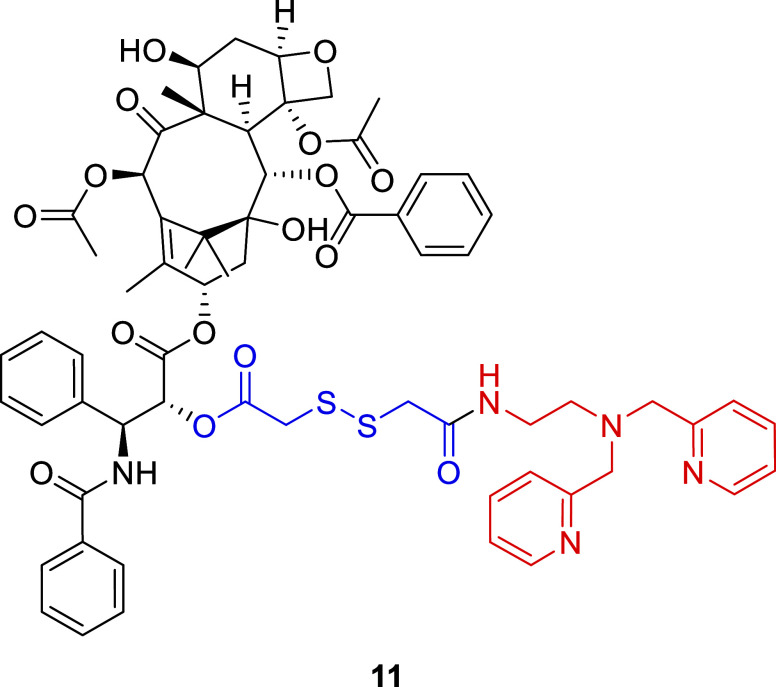
Structure of bifunctional compounds of type **11**. Highlighted in red the copper chelating moiety, in blue the redox-responsive linker, in black paclitaxel.

## 6 Copper Ionophores as Cuproptosis Promoters

Recent evidence joining cellular copper overload to cuproptosis has encouraged research not only into the characterization of the molecular mechanisms of this process but also into the identification of structural requirements of copper ligands capable of inducing this unique kind of cell death. Historically, the relationship between copper binding and cytotoxicity has evolved. Early observations (2004) established that the potency of copper compounds in inducing apoptosis, mediated by specific proteasome inhibition rather than mere oxidative damage, was strictly dependent on their metal binding affinity.^57^ Subsequently, in 2011, Tardito et al. definitively demonstrated that copper compounds must function as ionophores to induce cytotoxicity unrelated to apoptosis via copper overload and started to define the structural features of such ligands.^65^ Unlike chelating agents, which sequester and remove metals from biological systems, ionophores transport metals across cellular membranes thereby increasing their intracellular concentration. Similarly to other metal transporters, an effective copper ionophore should feature specific physicochemical properties including selectivity, binding affinity, and lipophilicity.^104^ Since copper-ionophores must shuttle Cu­(II) into the cells, the ligand structure must favor binding this ion over other species. However, the binding affinity must be balanced as high affinity could prevent the release of the metal inside the cell, while low affinity impairs the initial capture and transport function.^105^ Once it enters the cell, the Cu­(II) bound to the ionophore needs to be reduced to Cu­(I) to be released, a process that at least in part is regulated by FDX1.^72^ Consequently, an ideal copper ionophore should also exhibit a low affinity for Cu­(I) allowing that other intracellular ligands with higher affinity for Cu­(I) to displace it. Lipophilicity is a critical determinant for cellular activity of copper ionophore. An adequate partition coefficient (logP) is essential. Highly hydrophilic molecules may fail to cross membranes, whereas excessive lipophilicity causes limited deliverability from the aqueous phase to the lipid bilayer and lower mobility within the membranes.^106,107^

Although many copper complexes with cytotoxic properties have been synthesized, detailed information regarding their precise mechanisms of action remains limited. Since cuproptosis is an emerging concept, few molecules have been fully characterized as specific promoters of this distinct cell death mechanism. As a matter of fact, to date, most cuproptosis studies have relied primarily on compounds **1** and **2** (Figure 5), molecules repositioned as antitumor agents and utilized in clinical trials despite their molecular mechanism for their anticancer activity initially being undefined. Therefore, there is little information regarding the comprehensive SARs required for ionophores to exert an effective cuproptotic effect. Moreover, the ligand itself may have intrinsic biological activities, making it complex to isolate its specific contribution as an ionophore to overall cytotoxicity. The following section presents a selection of the most representative copper ionophores identified as cuproptotic agents. The preclinical and clinical status of these compounds is summarized in Table 3.

**Table 3:** Selected Preclinical and Clinical Studies on Copper Ionophores Investigated as Cuproptosis-Promoting Agents^a^

compound	selected preclinical studies (mouse)	selected clinical studies (phase, code)
**1**	tumors of various origins^109,110^	platinum-resistant ovarian, tubal or peritoneal cancer (phase II, NCT00888615)
		refractory solid tumors in combination with paclitaxel (phase I, NCT00088114)
		metastatic melanoma (phase II, NCT00084214)
		melanoma (phase III, NCT00522834)
**2** ^b^	inflammatory breast cancer^124^	lung cancer (phase III, NCT00312819)
		gastric cancer (phase II, NCT03714555)
	breast cancer^126^	breast cancer (phase II, NCT03323346)
		glioblastoma (phase III, NCT02678975)
	breast cancer and hepatocellular carcinoma^128^	metastatic NSCLC (phase III, NCT00312819)
**27**	-	prostate cancer (phase II, NCT00054015)
		pancreatic cancer (phase II, NCT00085371)
		metastatic renal cell carcinoma (phase II, NCT00075660)
		cervical and vaginal cancers in combination with cisplatin (phase III, NCT02466971)
**28**	ovarian endometrioid adenocarcinoma, NSCLC and breast adenocarcinoma^141^	-
**30**	breast cancer^147^	-
**39**	B-cell lymphoma and ovarian cancer^154^	relapsed or refractory hematological malignancies (phase I, NCT00963495)
	prostate cancer^155^	
**40**	glioblastoma and ovarian cancer^162^	-
**41**	colorectal cancer^164^	-

a Selected preclinical studies are reported according to the disease context evaluated, whereas clinical studies are listed with trial phase and ClinicalTrials.gov identifier (NCT code).

b FDA-approved for treatment of alcohol dependence.

### 6.1 Elesclomol

Elesclomol (ES, **1**, Figures 5 and 11) is a molecule originally developed by Synta Pharmaceuticals for the treatment of metastatic melanoma. A high-throughput screening of a Synta’s compound library employing an Hsp70 induction assay and the sarcoma cell line MES-SA/Dx5 for cell-based assays, identified the diphenylmalonohydrazide **12** (Figure 11) as hit compound. Compound **12** exhibited moderate *in vitro* activity inducing Hsp70 (EC_50_ = 0.75 μM) but potent antiproliferative activity against MES-SA/Dx5 cancer cell line (IC_50_ = 50 nM). Since compound **12** was found to be chemically and metabolically unstable, a collection of related analogues (compounds of type **13**, Figure 11) was synthesized to explore the SARs and optimize chemical properties and activity. These efforts led to **1** (formerly STA-4783), which showed anticancer activity across a wide range of tumor cell lines while unaffecting normal cell.^108^ Furthermore, **1** enhanced the efficacy of microtubule stabilizers, such as paclitaxel and docetaxel, in multiple human tumor xenograft mouse models.^109,110^

**Figure 11:**
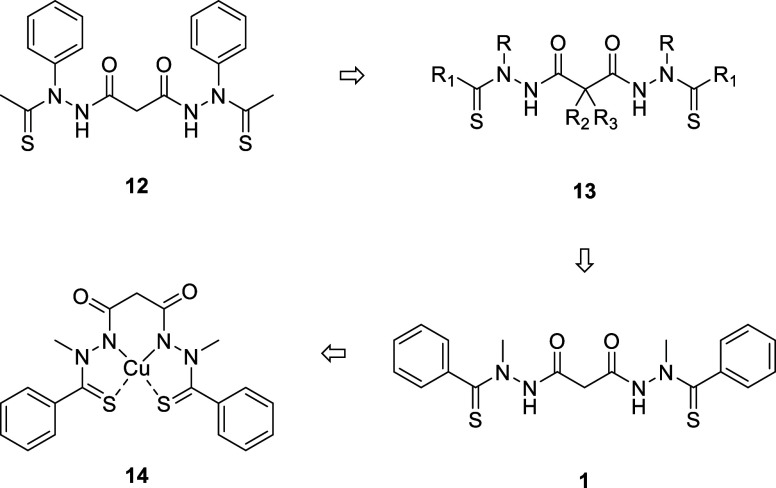
Structures of diphenylmalonohydrazide **12** and its analogues of type **13**; structures of elesclomol **1** and its copper complex **14**.

Synta Pharmaceuticals presented these results in 2007 and proposed a mechanism of action linked to ROS production. They claimed that **1** increased ROS levels and induced a strong heat-shock response, therefore selectively triggering apoptosis in cancer cells.^111,112^ However, it was not until 2012 that the cytotoxic activity of **1** was established to rely primarily on its copper-chelating ability.^113^ Compound **1** chelates extracellular Cu­(II) forming a highly cell permeable complex (**14**, Figure 11) that, after cellular uptake, rapidly and selectively targets the mitochondria. Inside the organelle, the reduction of Cu­(II) to Cu­(I), primarily in an FDX1-dependent manner,^72^ promotes copper-dependent cell death.

This occurs not merely through ROS generation but by targeting lipoylated and Fe–S cluster proteins, as recently proposed. After dissociation from the complex, **1** effluxes into the extracellular space to bind available Cu­(II) ions.

This process allows **1** to act as a copper ionophore shuttle, leading to the accumulation of copper within the mitochondria.^113^ Compound **1** was evaluated as sodium salt formulation in several clinical trials, both as a monotherapy and in combination with paclitaxel for treating solid tumors (ovarian, tubal, peritoneal, soft tissue sarcomas, and melanoma).^114–116^ Altogether, clinical results indicated that **1** has favorable safety profile but failed to produce the desired clinical response. Notably, Phase III results in patients with advanced melanoma confirmed that the addition of **1** to paclitaxel did not significantly improve progression-free survival. However, retrospective analysis suggested that lactate dehydrogenase (LDH) can be a predictive factor. As a matter of fact, patients with low or normal LDH levels showed a statistically significant better clinical outcome. These clinical results are consistent with recent findings on the association between the anticancer activity of **1** and cellular metabolic state. In fact, low LDH reflects a higher cellular dependency on mitochondrial metabolism rather than glycolysis, making these cells more susceptible to cuproptosis.^117^ These findings have revitalized research into mechanism-focused patient selection for future trials. The ability of **1** to deliver copper to mitochondria independently of copper transport machinery has been recently repurposed as treatment for MD. In mouse models, the Cu­(II)**-1** complex (**14**, Figure 11), but not free **1**, alleviated mortality and prevented detrimental neurodegenerative changes.^118^ In 2023, complex **14** was designated as an orphan medicine for the treatment of MD by the European Medicines Agency.

Compound **1** binds Cu­(II) with an extremely high affinity (log stability constant of 24.2), forming a stable 1:1 complex by losing two protons from the N1 and N2 atoms and donating four lone pairs (Figure 11). This increases the hydrophobicity of complex **14**. The X-ray crystallography reveals that coordination of nitrogen and sulfur atoms around the Cu­(II) atom results in an approximately square planar geometry. Selectivity is also high as, for instance, Fe­(III) and Fe­(II) have low affinity for **1**. Regarding its redox properties, it is noteworthy that CuCl_2_ oxidizes ascorbic acid 14.6-fold faster than complex **14**.^119^

The primary clinical limitation of **1** is the rapid elimination from plasma, with mean half-life values ranging from 0.79 to 1.06 h and the mean clearance of **1** ranges from 28.6 to 38.7 L/h/m^2^, making it difficult to maintain therapeutic concentration in patients. To overcome this hindrance, research is mainly focused on the development of nanoformulations to improve pharmacokinetics and therapeutic outcomes^120^ rather than manipulating the structure of **1**. Development of further analogues of **1** has been limited (Figure 12).^66^ SARs corroborated that the sulfur atoms of **1** are critical for activity, as substitution of one sulfur atom with oxygen (**15**) reduced the activity of the molecule by 300-fold and substitution of both sulfur atoms (**16**) completely inhibited the cytotoxic activity. Conversely, the replacement of the phenyl ring (compounds **17–20**) did not result in a dramatic change in the cytotoxic activity. Unfortunately, no data is currently available regarding the physicochemical properties or stability of these compounds.

**Figure 12:**
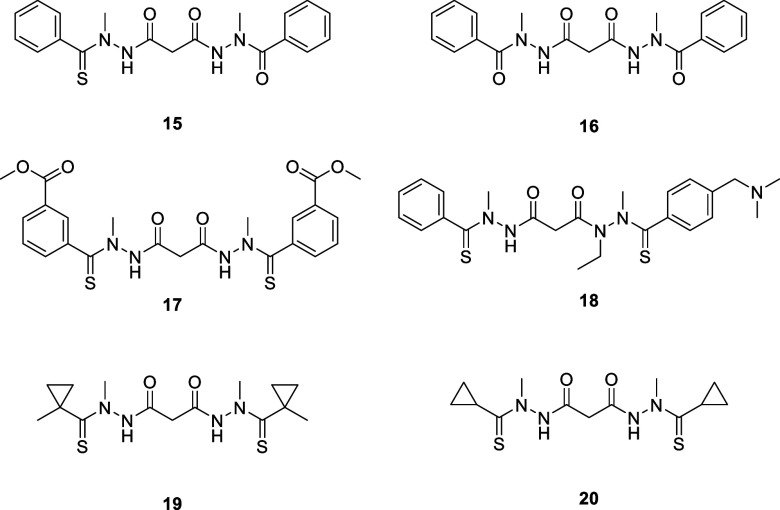
Structures of analogues of elesclomol **15–20**.

### 6.2 Disulfiram

Disulfiram (**2**, Figures 5 and 13) has been utilized for the treatment of alcohol dependence for more than 70 years. It irreversibly inhibits aldehyde dehydrogenase, resulting in the accumulation of acetaldehyde, which causes severe physical reactions upon alcohol consumption. **2** has been well tolerated by thousands of patients and, as an FDA-approved medication, has been repurposed for treatment of several diseases including cancer.^121^ In the last two decades, several clinical trials evaluated the efficacy of **2** in combination therapies in various cancers (lung, gastric, breast, glioblastoma),^122^ with the best results obtained for metastatic Non-Small Cell Lung Cancer (NSCLC).^123^ This evidence supported the role of **2** as a promising drug candidate for cancer therapy and has prompted research into its molecular mechanisms for clinical implementation.

**Figure 13:**
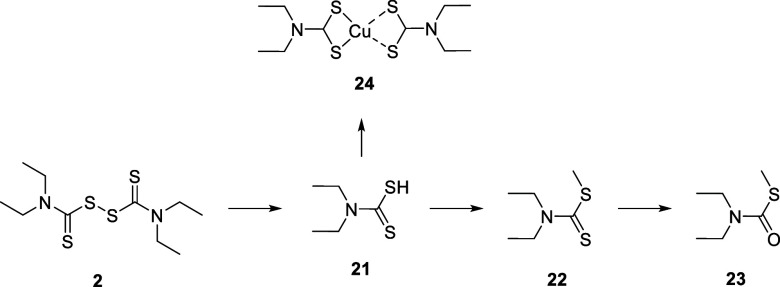
Structure of disulfiram (**2**), its metabolites (**21, 22** and **23**), and the copper complex of **21** (**24**).

Chemically, **2** is a disulfide derivative that rapidly reduces to two molecules of diethyldithiocarbamic acid (**21**, Figure 13) which is unstable and decomposes into diethylamine and carbon disulfide. However, **21** can also undergo methylation and subsequent oxidation to form various thiocarbamate such as methyl diethyldithiocarbamate (**22**, Figure 13) and methyl diethylthiocarbamate (**23**, Figure 13). Alternatively, **21** acts as a chelator, binding divalent metal ions such as copper and zinc to form stable, hydrophobic complexes. Cu­(II)-**21** complex (**24**, Figure 13) exhibit significantly higher stability (log *K* = 19) compared to zinc complexes (log *K* = 10). Substantial evidence indicates that, in the presence of Cu­(II), **2** rapidly form complex **24** also *in vivo*. *In vitro* and *in vivo* studies have demonstrated that **2** binds preferentially to Cu­(II) and this ability correlates with its anticancer activity, confirming that **24**, the active metabolite of **2**, acts as a copper ionophore.^124,125^ Indeed, the anticancer activity of **2** is dependent on the presence of Cu­(II) ions. In addition, **2** facilitates copper intracellular accumulation via a mechanism independent of the cellular CTR1 transporter.

Historically, the detailed mechanism of the anticancer activity of **2** remained unclear. Early hypotheses suggested that **2** triggers apoptosis by inducing oxidative stress^124^ or inhibiting proteasome activity in tumor cells.^126^ In 2017 Skrott et al. demonstrated that complex **24** does not directly inhibit 20S or 26S proteasome, but it acts as an indirect inhibitor of proteasome activity by targeting NPL4 protein (Nuclear Protein Localization protein 4), a protein essential for linking ubiquitinated proteins to the ubiquitin-proteasome system (UPS).^125^ The role of **21** as Cu­(II) ionophore in triggering cuproptosis was established in the pioneering works of Tsvetkov.^9,66^ As mentioned previously, **2** shares similar activities with **1**, and both compounds serve as valuable probes for study cuproptosis. Similar to **1**, the cytotoxic effect of **2** is dependent on the metabolic state of cells, with a loss of killing activity observed when cells were grown under glycolytic conditions. Additionally, FDX1 deletion results in resistance to cell death induced by **2**. These findings strongly supported the investigation of **2** and complex **24** as anticancer agents.

Despite these promising findings, clinical studies of **2** in cancer patients have not consistently replicated the excellent results obtained *in vitro* and *in vivo*. These mixed outcomes may be related to the rapid degradation of **2**, leading to insufficient concentration of active complex **24** in tumor tissues.^127^ On the other hand, the direct administration of the hydrophobic **24** complex is hampered by its very low aqueous solubility. To address these issues, several delivery systems for **2** or **24** have emerged in recent years.^128^ Another approach to overcome the poor pharmacokinetic properties of **2** and **24** involves the synthesis of analogues with improved physicochemical properties. However, although several compounds have been prepared, the only derivative that completely shares the activity and mechanism of action of **2** is the pyrrolidine dithiocarbamate **25** (PDTC, Figure 14).^129,130^ This is not unexpected as in **25** the *N*,*N*-diethyl substituention of **21** has been constrained in a pyrrolidine ring. The less flexible structure renders **25** far more stable at physiological pH, allowing it to reach the tumor site more effectively in laboratory models. As with **1**, even before the term cuproptosis was coined, several researchers observed that Cu­(II)-**25** complex (**26**, Figure 14) can induce cell death in various cell types by increasing intracellular copper levels. The cytotoxic activity was primary attributed to the inhibition of proteasomal activity, which subsequently induces apoptosis.^130–132^

**Figure 14:**
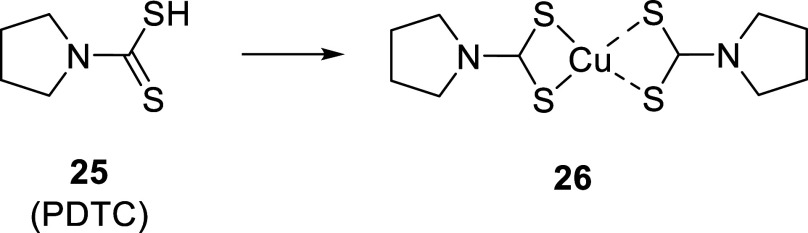
Structures of pyrrolidine dithiocarbamate (PDTC, **25**) and its copper complex **26**.

### 6.3 Thiosemicarbazones

Thiosemicarbazones are excellent metal chelators able to bind several transition metals such as iron, copper and zinc. Several copper mono and bis–thiosemicarbazone complexes have been prepared and evaluated for their cytotoxic activity.^133–135^ Typically, thiosemicarbazones coordinate the metal through the azomethine nitrogen and the sulfur atom, forming a thermodynamically stable five-membered ring. The incorporation of nitrogen-containing aromatic groups generates a tridentate ligand with the heterocyclic nitrogen becoming a third donor atom. This coordination results in two fused five-membered rings rather than just one, thereby increasing the stability of the complex. Notably, α-pyridyl thiosemicarbazones (Figure 15) are widely reported in the literature, and both the free ligands and their complexes have shown a broad spectrum of biological activity, including anticancer effects.^136,137^ This scaffold is highly versatile because varying the substituents tunes several physicochemical properties, such as chelating ability and lipophilicity. The latter, also in this class of compounds, correlates with antiproliferative activity.^138^

**Figure 15:**
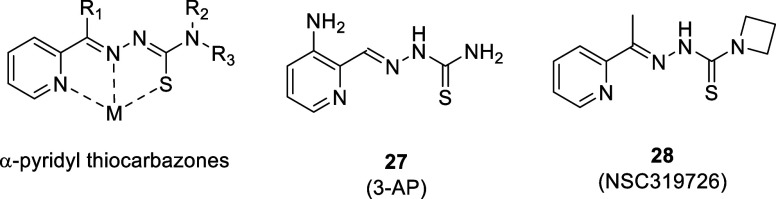
General structures of α-pyridyl thiosemicarbazones metal complexes, structures of triapine (3-AP, **27**) and NSC319726 (**28**).

Triapine (3-AP, **27**, Figure 15), the most intensively investigated ligand of this class, has been in clinical trials for several years in combination with other therapies against various cancers and recent randomized Phase III trial studies have evaluated its effectiveness. Compound **27** acts mainly as an inhibitor of ribonucleotide reductase (RNR), an enzyme involved in the synthesis of deoxyribonucleoside triphosphates essential for DNA replication and repair. The molecular mechanism of inhibition relies on the ability of **27** to strongly chelate iron in the cell. Specifically, the formation of the iron-**27** complex sequesters the iron required by RNR, leading to enzyme inactivation.^139^

While **27** is clinically defined by its iron-chelating properties, it serves as essential structural scaffold for this class of ligands; notably, the exact metal ion partners relevant to the biological activities remains a subject of investigation for many closely related analogues, several of which function primarily as copper ionophores.^140^ In the search for therapies targeting p53 mutant tumors, an in silico screening identified the pyridyl thiosemicarbazone NSC319726 (also known as Zinc Metallochaperone-1, ZMC1, **28**, Figure 15) as a compound able to reactivate mutant p53 by restoring the normal function of the most common p53 missense mutant. Initial mechanistic studies attributed this p53 reactivation to the compound’s role as a zinc ionophore, providing the intracellular deliver of zinc ions required to refold the misfolded protein to its wild-type conformation, thereby triggering apoptotic cell death in cancer cells.^141,142^ However, 2018 studies utilizing pharmacogenomic and molecular approaches in several primary-tumor-derived glioblastoma models revised this interpretation. Indeed, it was demonstrated that the cytotoxic activity of **28** does not involve known regulated cell death phenotypes, such as apoptosis or necroptosis, but rather it depends on the compound’s ability to bind copper.^143^ These findings were subsequently confirmed, as, even in the context of reactivation of missense p53 mutants, the copper-binding property of **28** is relevant for triggering the p53-mediated apoptotic signal. The primary role of copper, rather than zinc, in the anticancer mechanism of **28** is supported by the compound’s respective affinity constants with a 10^8^-fold preference for copper over zinc. Because of this extreme difference in affinity between the two ions, **28** preferentially binds copper first in cells and it acts as a zinc metallochaperone only after the available copper pool is exhausted.^144^

However, this earlier research relates the cytotoxic activity of **28** merely to generic copper-mediated oxidative stress. It was the pioneering 2022 Science paper^9^ that identified compound **28**, together with **1** and **2**, as key copper ionophores able to trigger cuproptosis. Consistent with this distinct cell death pathway, toxicity induced by **28** is blocked by copper chelators but not by apoptosis inhibitors. These studies highlighted the potential of **28** as a promising anticancer candidate and stimulated the synthesis and biological evaluation of pyridyl thiosemicarbazone-copper complexes as inducers of cuproptosis in cancer cells.^145^

### 6.4 Salicylaldehyde Hydrazones

Salicylaldehyde hydrazones are monobasic tridentate ligands capable of coordinating transition metals such as copper and iron. Coordination with copper affords 1:1 Cu­(II) complexes in which the metal ion is chelated by the deprotonated phenolic oxygen, the carbonyl oxygen, and the azomethine nitrogen atoms, while an additional anion (e.g., chloride) completes the coordination sphere (Figure 16). Recently, several derivatives of this class have been characterized and evaluated as copper ionophores able to induce cuproptosis.

**Figure 16:**
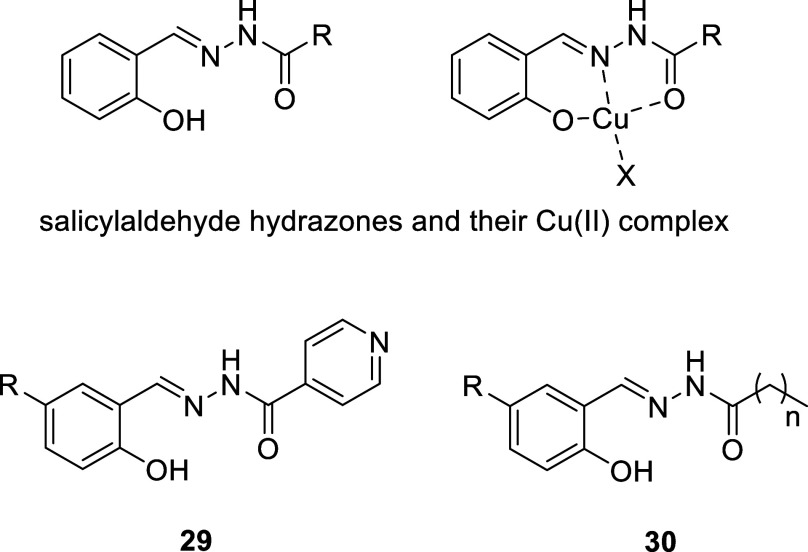
General structure of salicylaldehyde hydrazones and their copper complexes, structures of salicylaldehyde isonicotinoyl hydrazones (**29**, R = H, NO_2_, OMe) and alkyl derivatives (**30**, *n* = 0, 2, 4, 6, 8).

In 2018, a small series of salicylaldehyde isonicotinoyl hydrazones of type **29** (Figure 16) were synthesized, chemically characterized, and evaluated for copper-dependent cytotoxic activity. The nature of the substituent strongly affected the chelation ability of the ligand. As anticipated, the highest stability constant (*K* = 2.28 × 10^4^ M^–1^) was observed for the nitro-substituted derivative, whose electron-withdrawing effect significantly increases the acidity of the phenolic group compared with the unsubstituted (*K* = 5.26 × 10^2^ M^–1^) and methoxyl (*K* = 1.20 × 10^3^ M^–1^) analogues. Although not assessed, a similar trend could reasonably be expected for the coordination of other metals, such as iron. In contrast, the substituent appeared to exert little influence on compound lipophilicity, with calculated logP values of approximately 2.1 for the free ligands and between 0.47 and 0.72 for the corresponding copper complexes. When tested for cytotoxicity, the ligands alone did not significantly affect the viability of human hepatoma HepG2 cells while the cotreatment with Cu­(II) induced cell death. The cytotoxicity activity of derivatives correlates with their ability to induce copper accumulation in cells, with the unsubstituted derivative emerging as the most active compound.^146^

More recently, a collection of salicylaldehyde hydrazone analogues (**30**, Figure 16) was investigated for ion transport efficiency and cytotoxic activity. In these derivatives, the pyridine ring present in compounds **29** was replaced by *N*-alkyl chains of different lengths. Alkyl length has a moderate effect on the chelation ability of the ligands, with binding constants falling between 4.04–11.23 × 10^4^ M^–1^ for copper. Although these ligands showed high selectivity for Cu­(II) over other divalent ions such as zinc, calcium and magnesium, they displayed comparable affinity toward iron. Conversely, the experimental log *P* values showed a strong positive correlation with alkyl chain length for both the free ligands and their copper complexes, the latter generally being more lipophilic (log *P* from −1.05 to 0.97) than the corresponding ligands (log *P* from −0.60 to 0.42). These ligands alone increased intracellular copper levels and induced cytotoxicity on triple-negative breast cancer cell lines. Moreover, the antitumor activity was markedly enhanced by introducing Cu­(II) in the medium, whereas the addition of a copper chelator attenuated cell death, thereby confirming the ionophore nature of these compounds. Notably, for the Cu­(II) complexes, a strong correlation between IC_50_ values and lipophilicity was observed, consistent with findings reported for other classes of copper ionophores. The complex bearing a five-carbon alkyl chain (*n* = 4), featuring an intermediate lipophilicity (log *P* = 0.53), displayed the highest activity, whereas both more hydrophilic and more hydrophobic analogues were less effective. Mechanistic studies demonstrated that cell death induced by compound **30** (*n* = 4) occurs through cuproptosis. Indeed, toxicity induced by compound **30** (*n* = 4) cannot be rescued by inhibitors of apoptosis, autophagy, or necroptosis. In addition, the complex downregulated the expression of FDX1, DLAT, and LIAS while promoting DLAT oligomerization. This derivative, when tested in mouse models of breast cancer in combination with copper, exhibited potent antitumor activity triggered by cuproptotic mechanism.^147^

Taken together, these findings further support the notion that the cellular outcome for copper ionophore is strongly dependent on a proper chelation ability and an optimal lipophilicity window.

### 6.5 Pyrazole-Pyridine and Bis-Pyrazole Ligands

As previously mentioned, Tardito et al. correlate the nonapoptotic cell death induced by copper-binding agents with the ability of the ligand to act as an ionophore for the metal ion.^65^ For their studies, a collection of pyrazole-pyridine (**31–33**, Figure 17) and bis-pyrazole ligands (**34–36**, Figure 17) was synthesized, and the corresponding Cu­(II) complexes were prepared and characterized. The presence of different alkyl substituents and a sulfur atom affect both the coordinative ability and hydrophobicity of the compounds. The stability constants (log *K*) of these compounds ranged between 3.34 and 4.69. The highest values were found for compounds featuring sulfur in a sterically favorable position, which allowed the exploitation of three donor atoms (N,N,S) for copper binding rather than the bidentate *N*,*N*-coordination. These values indicate that ligands are mostly dissociated from copper in the extracellular environment, where copper is sequestered by binding to cellular competitors such as amino acids. For instance, the first and the second stability constants of the [Cu­(histidine)_2_] complex are 10.1 and 8.0 respectively,^148^ therefore several orders of magnitude higher than those of these pyrazole-based ligands. Conversely, the hydrophobicity of the compounds was modulated to cover a wide range of log *P* values. The cytotoxic activity of the complexes was evaluated in human fibrosarcoma HT1080 cells, revealing that increased cytotoxicity was associated not with the higher stability of the complex, but rather with its hydrophobicity. The relationship between the calculated log *P* value of the ligand and the cytotoxic activity of the respective complex suggests that the optimal logP value range is between 4 and 6. These findings were later corroborated by the same group and others regarding different classes of compounds.^149^ Moreover, the strong correlation between cytotoxic activity, intracellular copper content, and the activation of caspase-independent cell death mechanism, led the authors to classify their bis-pyrazole and pyrazole-pyridine ligands as copper ionophores.^65^

**Figure 17:**
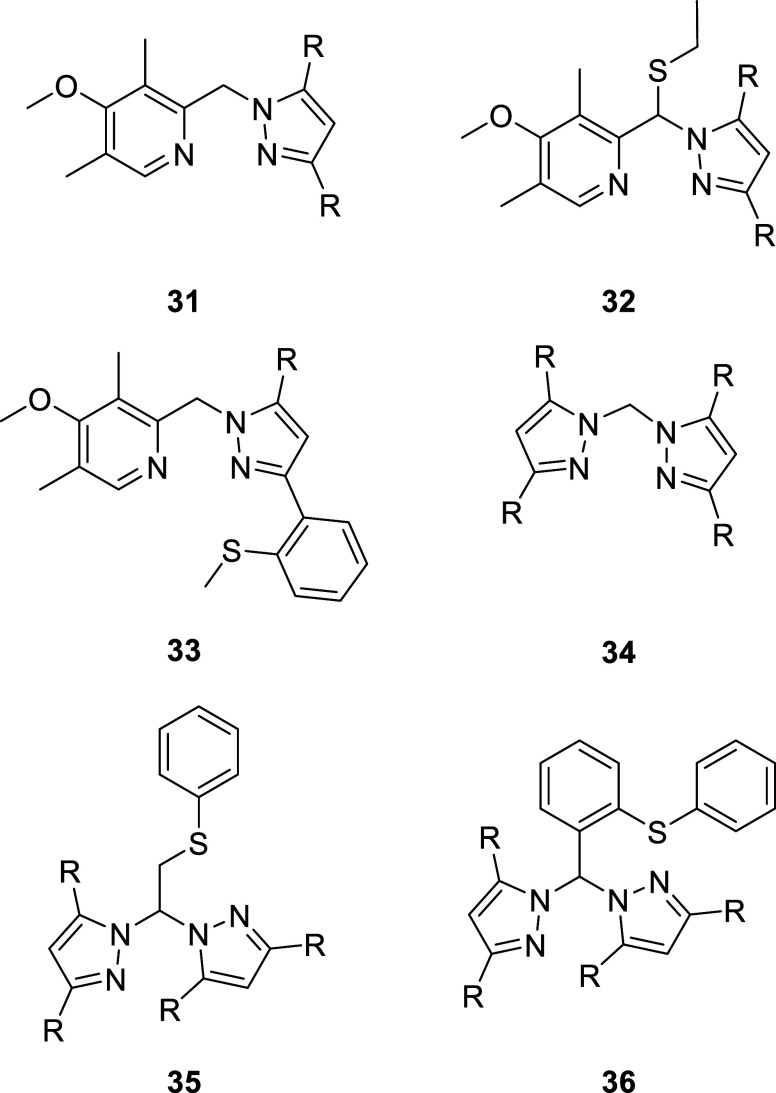
General structures of pyrazole-pyridine (**31–33**) and bis-pyrazole (**34–36**) ligands; R = H, alkyl or thioalkyl group.

### 6.6 8-Hydroxyquinoline Derivatives

8-Hydroxyquinoline (8-HQ) derivatives (compounds of type **37**, Figure 18) are lipophilic ligands characterized by the presence of a N,O-binding system that is suitable for interacting with divalent metal ions (complexes of type **38**, Figure 18) and have been extensively investigated as potential therapeutic agents for the treatment of several diseases.^150^ A well-known example is 5-chloro-7-iodo-8-hydroxyquinoline (clioquinol, CQ, **39**, Figure 18) which was used for decades as antimicrobial, antifungal and antiprotozoal agent. However, oral use of **39** has been discontinued or severely restricted because of its neurotoxicity.^151^ 8-HQ derivatives are able to redistribute metals into cells and, therefore, these compounds have been characterized as copper and zinc ionophores and gained attention as candidate drugs for the treatment of some neurological diseases (e.g., Alzheimer’s disease^152^ and Huntington’s disease^153^) and cancers. As regards anticancer activity, research into the cytotoxic properties of 8-HQ derivatives has increasingly focused on their copper-shuttling properties and, consequently, on the ability to induce cuproptosis.

**Figure 18:**
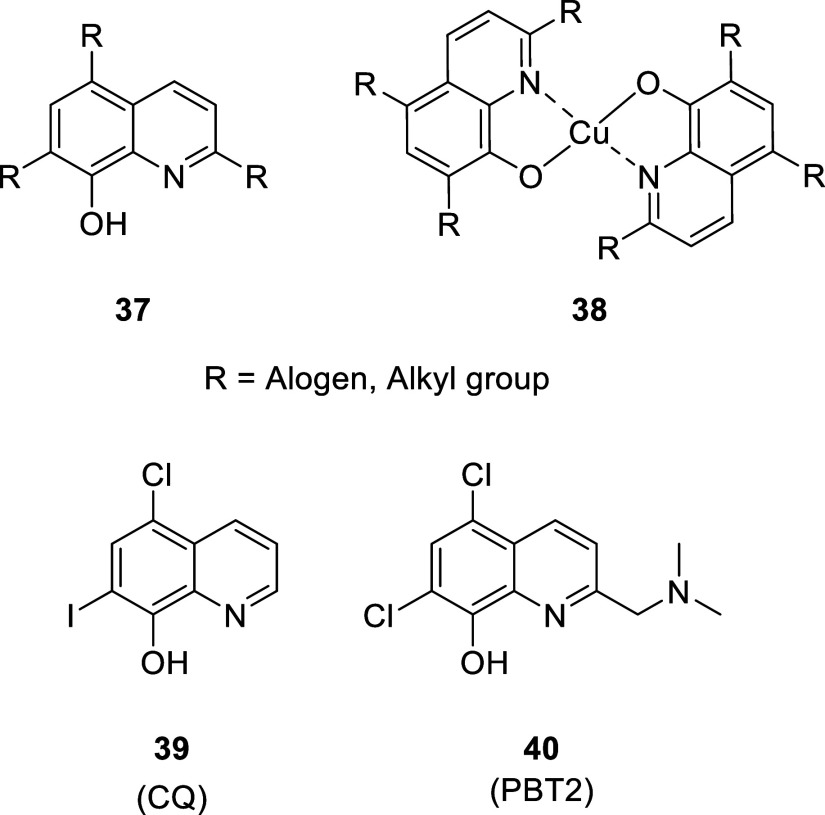
General structure of 8-hydroxyquinoline (8-HQ) derivatives (**37**) and their copper complexes (**38**); structures of clioquinol (CQ, **39**) and PBT2 (**40**).

In the early 2000s several authors initially attributed the capability of 8-HQ derivatives to induce apoptosis to proteasome inhibition in a copper-dependent manner not related to oxidative effects.^57,129^ The first reports clearly associating the anticancer activities of 8-HQ derivatives with their aptitude to act as metal ionophores emerged in 2005. Investigating the mechanism behind the ability of **39** to induce cytotoxicity in several representative human cancer cell lines and to inhibit tumor growth in mouse xenografts, Ding et al. disproved the hypothesis that the compound acted merely as a chelator. Indeed, they demonstrated that supplementation with copper, iron or zinc did not overcome the cytotoxicity induced by **39** but conversely enhanced it. Further experiments led them to conclude that **39** exert antineoplastic activity by transporting metals intracellularly, therefore acting as an ionophore. While mitochondria were identified as a key site of metal injury, the cytotoxic activity was attributed to increased intracellular zinc concentration and caspase-dependent apoptosis rather than copper overload.^154^ Two years later Chen et al. proposed an alternative mechanism primarily involving copper. They suggested that **39** reacts with endogenous copper within tumor tissue and forms an active complex that exhibits antitumor activity, which requires proteasome inhibition.^155^ Further studies confirmed that the toxicity of **39** correlates with the level of extracellular copper and that it can be abrogated by using copper chelators, therefore supporting the role of **39** in causing intracellular copper overload with subsequent toxicity.^156^ Finally, in 2012, the work of Tardito et al. confirmed that the cytotoxic activity of 8-HQ derivatives was linked with their ionophoric nature. Remarkably, they pointed out that the ligand-dependent copper accumulation triggers a nonapoptotic cell death, paving the way for the discovery of cuproptosis.^149^ Compound **39** entered Phase I clinical trials for the treatment of relapsed or refractory hematological malignancies, though it was not studied further due to lack of recognized clinical responses and reported side effects associated with neuropathy. Other 8-HQ derivatives have been studied as anticancer agents. For instance, 5,7-dichloro-2-[(dimethylamino)­methyl]-8-hydroxyquinoline (PBT2, **40**, Figure 18) was found to be more cytotoxic than other 8-HQ derivatives, despite featuring inferior ionophoric capability. This suggests that **40** acts with a different mechanism of action or alters cellular copper distribution differently than **39** ^157^ and it can be related to its different coordination chemistry (see below). Compound **40** entered clinical trials for the treatment of neurological diseases such as Alzheimer’s disease and Huntington’s disease.

Chemically, 8-HQ derivatives are planar, N,O-donating ligands that have a high affinity for divalent ions such as copper, zinc and iron. In general, affinity for copper is significantly higher than for other divalent cations. For instance, the affinity constant calculated for 1:2 stoichiometry of Cu­(II)-**39** (1.2 × 10^10^) is at least an order of magnitude higher than for Zn­(II) (7.0 × 10^8^).^158^ Typically, the complex forms with a 1:2 stoichiometry (one metal ion for every two 8-HQ molecules). The ligands are arranged in a trans geometry, with the nitrogen and oxygen atoms on the opposite sides with respect to the metal center (**38**, Figure 18). 8-HQ and 5,7-dihalogenated 8-HQ derivatives bind Cu­(II) in a square planar-like geometry with two bidentate 8-HQ ligands. On the other hand, substituents in position 2 can significantly alter the structural aspects of the complex, including planarity and coordination geometry. For instance, **40** can involve the exocyclic dimethylamine in the coordination site, therefore acting as a bi- or tridentate ligand. This variability has a high impact on stoichiometry, affinity for divalent ions and geometry of the complex. Due to this lack of selectivity and high versatility, **40** can form several coexisting complexes with Cu­(II) and Zn­(II) depending on the solvent and, *in vivo*, depending on the presence of competitive ligands.^159,160^ These properties can explain the distinct biological outcomes of compound **40**. The chemical features described above must be taken into account when evaluating the biological effects of these ionophores. In addition, decorating the 8-HQ scaffold with substituents tunes not only the coordinative ability but also the hydrophobicity, which impacts membrane permeability and therefore the biological outcomes. It has been proven that the highest activity is obtained with complexes of ligands featuring intermediate lipophilicity (log *P* between 1.5 and 3). The more lipophilic derivatives show reduced potency whereas the more hydrophilic derivatives display no cytotoxic activity when complexed with Cu­(II).^149^ On the other hand, a major challenge is the low aqueous solubility of these complexes which troubles their characterization and *in vitro* studies and, therefore, hindering their development as drug candidates.^161^ Current research is focused on the design of more soluble molecules together with the development of suitable formulations to overcome these limitations.^162^

### 6.7 Natural Products. Imidazole-Based Compounds: Chagosendine C

The screening of a library of marine products led to the isolation of natural imidazole-based homodimer complexes, termed chagosendines, from the sponge *Leucetta chagosensis*. Some of these compounds exhibited *in vitro* cytotoxic activity against various cancer cell lines (IC_50_ values ranging from 0.3 to 4.4 μM), displaying higher potency than either their free ligands or their corresponding nickel or zinc complexes.^163^

Subsequent studies revealed that one of them, Chagosendine C (**41**, Figure 19) significantly inhibited the growth of both HCT116 and RKO colorectal cancer cells (IC_50_ values of 0.79 μM and 1.12 μM, respectively) and was able to counteract oxaliplatin resistance in these cell lines. More importantly, the complex induces nonapoptotic-dependent cell death in a copper-dependent manner. Transcriptome sequencing analysis on RKO cells treated with **41** revealed an enrichment of genes related to misfolded protein binding, as well as cellular response to oxidative stress and heat. Additionally, **41** was found to disrupt mitochondrial function and significantly inhibit cellular respiration. By combining the drug affinity responsive target stability (DARTS) technique with knockout experiments and cellular thermal shift assay (CETSA), FDX1 was identified as direct target for **41**. Collectively, these findings support the conclusion that the molecular mechanisms underlying **41** induced cell death involves cuproptosis. Mouse models of colorectal cancer further support the potential of **41** as a drug candidate, as it suppressed tumor growth without detectable toxic effects.^164^

**Figure 19:**
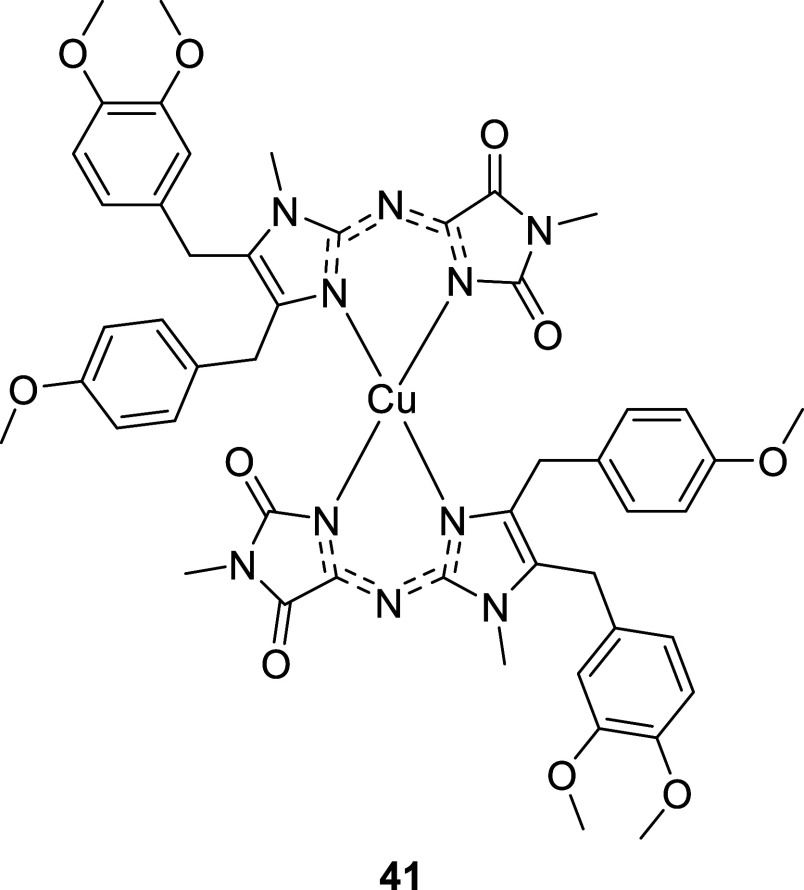
Structure of alkaloid Chagosendine C (**41**).

## 7 Conclusions and Perspectives

Copper is an essential micronutrient that plays significant roles in numerous biological functions, and its cellular content is finely regulated. Imbalances in copper homeostasis are associated with several diseases, including cancer. Historically, it has been known that copper concentration in cells can modulate tumor onset and proliferation. Indeed, increases in cellular copper uptake have exhibited both pro- and anticancer properties in various contexts. On one hand, cancer cells have a much higher demand for copper than normal cells to sustain growth and proliferation. Conversely, copper accumulation can induce oxidative stress and cell death. On this basis, over the years, several ligands capable of modulating cellular copper concentration have been studied as antitumor agents. While several ligands have entered clinical trials combined with standard therapies, none have yet achieved regulatory approval as anticancer agent. This lack of success may be ascribed to several issues including the incomplete understanding of the molecular mechanisms behind the role of copper homeostasis across various cancers. As a matter of fact, only in the past few years there has been an important step forward in the field with the clear definition of cuproplasia, indicating the copper-driven cellular growth and proliferation, and cuproptosis, referring to a copper-dependent form of regulated cell death. As a result, the development of copper ligands able to manipulate cellular copper levels has garnered increasing interest as an innovative approach to cancer therapy. Manipulation of cellular copper levels can be achieved using chelators, to lower levels and counteract cuproplasia, or ionophores to elevate levels and promote cuproptosis. Regarding copper chelators, research has largely relied on repurposing drugs originally designed for copper accumulation disorders. Their failure in clinical outcomes is mainly related to pharmacokinetic issues, such as short half-lives, and nonspecific toxicity. Current research is mainly focused on incorporating these small molecules into bifunctional compounds or into stable long-circulating macromolecular or delivery systems exploiting nanotechnology. As far as copper ionophores able to induce cuproptosis are concerned, the development of small molecules is still lagging behind. Most mechanistic studies rely on elesclomol (**1**) and disulfiram (**2**), molecules discovered serendipitously rather than through rational design. Despite the preparation of several copper ligands and complexes with cytotoxic activity, these studies were conducted prior to the recognition of cuproptosis, and these compounds exhibited undefined or multifaceted mechanisms of action. Therefore, to date, there are no robust SARs regarding this specific mechanism of regulated cell death. Nevertheless, recent advances in the exploration of mechanisms of cuproptosis regulation combined with earlier medicinal chemistry data on collection of copper binding compounds highlight the essential physicochemical properties that should be taken into account to develop copper ionophores for use as molecular probes to detect cuproptosis and as potential anticancer agents. First, as the reduction of Cu­(II) to Cu­(I) is pivotal for cuproptosis, the redox properties of the copper center should be evaluated. Since Cu­(I) release outside of mitochondria can occur in both an FDX1-dependent and independent manner, copper ionophores should be designed to maximize interaction with FDX1 or promote self-reduction of Cu­(II) within the intracellular environment. Second, the stability constant should be optimized to be selective for Cu­(II) binding over Cu­(I) and other divalent metals, such as zinc or iron, and tuned to ensure the delivery of copper to the tumor site. Finally, the lipophilicity of the ligand and its Cu­(II) complex should be adjusted to provide high intracellular copper accumulation. To this purpose, the determination of cellular copper content after compound treatment could be highly valuable.

In parallel with the design of copper ionophores, biological evaluation of their activity should consider several aspects. Because mitochondrial respiration enhances sensitivity to cuproptosis, the estimation of cytotoxic activity must take into great consideration the cell line used and its metabolic state. For instance, in MCF7 cells, the **1**-Cu­(II) complex (**14**) is up to 1000-fold more potent in galactose-containing media (mitochondria respiration-dependent) compared to glucose-containing media (glycolysis-dependent). Moreover, evaluating the ability of the Cu­(II)-complexes to induce oligomerization of lipoylated DLAT is crucial to detect cuproptosis.

The *in vivo* outcome of copper modulation is context-dependent and the tumor microenvironment (TME) has to be considered. Emerging evidence highlights a potent link between copper availability and immune evasion, notably through the induction of PD-L1 expression, which suggests that copper chelation could act as a sensitizer for checkpoint inhibitor therapies. Finally, increasing evidence suggests that copper can modulate HIF1α activity under the hypoxic conditions of tumor microenvironment, which in turn is able to drive resistance to cuproptosis and to support tumor progression and metastatic dissemination. In this context, copper chelators and ionophores may exert antitumor effects not only through direct cytotoxicity but also by reshaping the TME, modulating oxidative stress, mitochondrial metabolism, inflammatory signaling, and antitumor immune responses.

All these considerations have a significant impact on the clinical evaluation of both new copper ligands and molecules already in clinical trials. As the tumor metabolic profile correlates to copper ionophore sensitivity, clinical outcomes should be carefully reviewed. In this context, multiomics strategies will help in the identification of proper biomarkers for patient selection.

## Abbreviations

3-AP: triapine; 8-HQ, 8-hydroxyquinoline
AD: Alzheimer’s disease
ADP: adenosine diphosphate
AKT: protein kinase B
ATOX1: antioxidant 1 copper chaperone (or antioxidant protein 1)
ATP: adenosine triphosphate
ATP7A/B: copper-transporting P-type ATPase α/β
ATTM: ammonium tetrathiomolybdate
CcO: cytochrome *c* oxidase (or complex IV)
CCS: copper chaperone for superoxide dismutase 1
CETSA: cellular thermal shift assay
COX17: cytochrome *c* oxidase copper chaperone
CP: ceruloplasmin
CQ: clioquinol
CTR1: copper transport protein 1 (or copper transporter 1)
DARTS: drug affinity responsive target stability
DCT1: divalent-cation transporter 1
DLAT: dihydrolipoamide S-acetyltransferase
DMT1: divalent metal transporter 1
DPEN: d-penicillamine
DSF: disulfiram
EGFR: epidermal growth factor receptor
ERK1/2: extracellular signal-regulated kinases 1 and 2
ES: elesclomol
FDX1: ferredoxin 1
GPX4: glutathione peroxidase 4
GSH: glutathione
HIF1α: hypoxia-inducible factor 1-alpha
LDH: lactate dehydrogenase
LIAS: lipoic acid synthase
Lip: lipoyl moiety
MD: Menkes disease
MEK1/2: mitogen-activated protein kinase kinase
METTL16: methyltransferase-like 16
MTs: metallothioneins
NPL4 protein: nuclear protein localization protein 4
NSCLC: non-small cell lung cancer
PBT2: 5,7-dichloro-2-[(dimethylamino)­methyl]-8-hydroxyquinoline
PDC: pyruvate dehydrogenase complex
PDK1: 3-phosphoinositide-dependent protein kinase 1
PD-L1: programmed death-ligand 1
PDTC: pyrrolidine dithiocarbamate
PF: proangiogenic Factors
RNR: ribonucleotide reductase
ROS: reactive oxygen species
SA: serum albumin
SARs: structure–activity relationships
SCO1/2: synthesis of cytochrome *c* oxidase 1 and 2
SOD1: superoxide dismutase 1
STEAP: six-transmembrane epithelial antigen of the prostate
TCA: tricarboxylic acid
TEPA: tetraethylenepentamine
TETA: triethylenetetramine
TGN: trans-golgi network
TME: tumor microenvironment
TTM: tetrathiomolybdate
Ub: ubiquitin
UBE2D: ubiquitin-conjugating enzyme E2 D
ULK1/2: UNC-51 like autophagy activating kinase 1 and 2
UPS: ubiquitin-proteasome system
WD: Wilson disease
ZMC1: zinc metallochaperone-1.
